# Refining the Mouse Subtotal Nephrectomy in Male 129S2/SV Mice for Consistent Modeling of Progressive Kidney Disease With Renal Inflammation and Cardiac Dysfunction

**DOI:** 10.3389/fphys.2019.01365

**Published:** 2019-11-15

**Authors:** James O’Sullivan, Sarah Louise Finnie, Oliver Teenan, Carolynn Cairns, Andrew Boyd, Matthew A. Bailey, Adrian Thomson, Jeremy Hughes, Cécile Bénézech, Bryan Ronald Conway, Laura Denby

**Affiliations:** ^1^Centre for Cardiovascular Science, Queen’s Medical Research Centre, The University of Edinburgh, Edinburgh, United Kingdom; ^2^Centre for Inflammation, Queen’s Medical Research Centre, The University of Edinburgh, Edinburgh, United Kingdom

**Keywords:** chronic kidney disease (CKD), modeling disease, cardiac hypertrophy, renal fibrosis and inflammation, monocytes/macrophages

## Abstract

Chronic kidney disease (CKD) is prevalent worldwide and is associated with significant co-morbidities including cardiovascular disease (CVD). Traditionally, the subtotal nephrectomy (remnant kidney) experimental model has been performed in rats to model progressive renal disease. The model experimentally mimics CKD by reducing nephron number, resulting in renal insufficiency. Presently, there is a lack of translation of pre-clinical findings into successful clinical results. The pre-clinical nephrology field would benefit from reproducible progressive renal disease models in mice in order to avail of more widely available transgenics and experimental tools to dissect mechanisms of disease. Here we evaluate if a simplified single step subtotal nephrectomy (STNx) model performed in the 129S2/SV mouse can recapitulate the renal and cardiac changes observed in patients with CKD in a reproducible and robust way. The single step STNx surgery was well-tolerated and resulted in clinically relevant outcomes including hypertension, increased urinary albumin:creatinine ratio, and significantly increased serum creatinine, phosphate and urea. STNx mice developed significant left ventricular hypertrophy without reduced ejection fraction or cardiac fibrosis. Analysis of intra-renal inflammation revealed persistent recruitment of Ly6C^*hi*^ monocytes transitioning to pro-fibrotic inflammatory macrophages in STNx kidneys. Unlike 129S2/SV mice, C57BL/6 mice exhibited renal fibrosis without proteinuria, renal dysfunction, or cardiac pathology. Therefore, the 129S2/SV genetic background is susceptible to induction of progressive proteinuric renal disease and cardiac hypertrophy using our refined, single-step flank STNx method. This reproducible model could be used to study the systemic pathophysiological changes induced by CKD in the kidney and the heart, intra-renal inflammation and for testing new therapies for CKD.

## Introduction

Chronic kidney disease (CKD) is increasing in prevalence (Eckardt et al., 2013) and is a significant public health problem due to its associated economic burden (Kerr et al., 2012). Multiple clinical etiologies result in CKD with hypertension and diabetes being the leading causes (Horowitz et al., 2015; Alicic et al., 2017; Obrador and Levin, 2019). CKD progression is staged via estimated glomerular filtration rate (eGFR) and urinary albumin-creatinine ratio (ACR) (Stevens and Levin, 2013). Patients with CKD are at increased risk of cardiovascular disease (CVD) and this risk increases as renal function declines. Once patients reach end-stage kidney disease (ESKD) requiring dialysis or transplantation, the risk of CVD is 10–30x that of the general population with cardiovascular events accounting for almost 50% of deaths in CKD patients (Di et al., 2015).

The common end-pathway of progressive CKD is the deposition of fibrotic scar tissue that replaces the functional renal parenchyma in the form of tubulointerstitial fibrosis and glomerulosclerosis. The underlying mechanisms of fibrosis remain incompletely understood as it is a complex process involving a diverse array of cell types and molecular pathways, with cross-talk between cell types being evident (Gewin et al., 2017). These cell types include fibroblasts, tubular epithelial cells, macrophages, endothelial cells, dendritic cells, and lymphocytes (Boor et al., 2010). For example, fibroblasts differentiate into myofibroblasts, proliferate, and deposit extracellular matrix components (Mack and Yanagita, 2015). The sources of myofibroblasts within the injured kidney have been the subject of intense study, perivascular Gli^+^ progenitor cells have been suggested to be particularly important (Kramann et al., 2015). Another notable feature of CKD is tubular atrophy and loss of tubular epithelial cells (Venkatachalam et al., 2015; Schelling, 2016; Webster et al., 2017). The renal tubule has long been thought of as a target of renal injury, however it may also function as a propagator of injury as tubular cells may undergo cell-cycle arrest, de-differentiation, and acquire a pro-secretory phenotype (Gewin, 2018). Cytokines secreted by tubular cells may act as paracrine factors to promote the production of collagenous matrix by surrounding myofibroblasts (Gewin, 2018).

Subtotal nephrectomy, or 5/6 nephrectomy, is used as a rodent model of progressive CKD (Yang et al., 2010). Historically, the subtotal nephrectomy model was performed in rats, although it has more recently been conducted in mice (Ma and Fogo, 2003; Kennedy et al., 2008; Siedlecki et al., 2009; Yang et al., 2010; Gava et al., 2012; Oosterhuis et al., 2017). The effectiveness of STNx to produce experimental-CKD in mice has been found to vary depending on the strain of mouse used (Leelahavanichkul et al., 2010), with C57BL/6 mice being more resistant (Kren and Hostetter, 1999; Ma and Fogo, 2003; Leelahavanichkul et al., 2010) and SV129/CD1 mice being permissive (Ma and Fogo, 2003; Kennedy et al., 2008; Siedlecki et al., 2009; Leelahavanichkul et al., 2010). However, the results in mice have been inconsistent and there is a lack of technical information about how the model was performed, any power calculation data, mortality rates, information on post-surgery animal welfare and whether the ARRIVE guidelines were followed. This lack of standardization in the model in mice likely contributes to the inconsistencies reported (Chatzimanouil et al., 2018).

We sought with this paper to standardize the STNx model in mice, to improve animal welfare standards and define the renal and cardiac effects to enable consistent modeling of the pathophysiological changes induced during progressive CKD.

## Materials and Methods

### Single Step Flank Subtotal Nephrectomy Model

The refined STNx model involves a single anesthetic and surgery (∼40 min), performed via flank incisions that result in improved animal condition scores, reduced mortality with reproducible outcomes between studies.

Male 129S2/SV mice were obtained from Envigo and used when 6–8 weeks old (weighing 24.7 ± 0.37 g SEM). Male Gli1 × Ai14 mice on a C57BL/6 genetic background were used at 9 ± 3 weeks old (weighing 31.9 ± 1.1 g SEM). Mice were group-housed and provided with *ad lib* access to water and fed with Rm1 standard chow (Special Diets Services) with the following content 0.25% Na, 0.67% K, 0.38% Cl. Mice were also given environmental enrichment. A 12-h light–dark cycle was maintained. During the study, mice were weighed weekly and had their condition recorded. ARRIVE guidelines were adhered to at all times. Only male mice were selected as unlike in other organs, notable sex difference in myeloid cells, including macrophages have been documented in the kidney (Bain et al., 2016).

Animals were randomized to receive sham or subtotal nephrectomy surgery (STNx) using a random number generator website^1^. Prior to surgery, mice had a timed overnight urine sample collected (single housed metabolic cage), blood sample taken (superficial vein) and blood pressure measured (tail cuff). Immediately prior to surgery, mice were weighed and a total of four studies including two pilot studies were performed.

Surgery was performed in a sterile surgical environment using inhalational isoflurane for anesthesia. Once anesthetised, the mouse was shaved and received perioperative s.c. analgesia.

The mouse was initially placed on the left lateral side and an incision was made on the flank over the right kidney. The right kidney was located and carefully maneuvred out of the incision site. The adrenal gland was carefully blunt dissected away from the kidney to avoid adrenalectomy. The right renal pedicle was clamped and a nephrectomy performed. The vascular clamp was removed, the renal bed checked for signs of bleeding and the abdominal wall sutured closed and skin clips applied to close the outer skin incision.

The left kidney was then located and adrenal gland blunt-dissected away. The renal artery and vein were isolated and clamped ensuring ischemic time was less than 5 min. Renal poles (approximately 2/3 renal mass) were then surgically removed and spongostan applied. The vascular clamp was released and once hemostasis had been achieved, the kidney was placed back into the abdomen and the incisions closed. For sham surgery, animals were prepared the same way, had bilateral flank incisions and both kidneys isolated and manipulated.

At the end of surgery, mice were immediately placed in a fresh cage with littermates in a hotbox at 28°C, where they remained for 7 days. During this time, the mice were checked three times daily and scored using bespoke animal condition scoring sheets (Supplementary Table 1). After this time the mice were weighed, skin clips removed and placed in a regular animal holding room and maintained under normal conditions.

For quality control purposes, the weight of kidney removed was calculated to estimate how much residual kidney was left (with the caveat that the kidneys have a small mismatch in weights). The weight of the whole right kidney was measured and compared to the weight of the two pole sections of kidney removed from the left kidney. In order to maintain consistency in this model we ensured that the percentage remaining was as consistent as possible. Residual kidney mass was calculated by the following:

% left renal mass remaining = 100 – [(left kidney sections weight/whole right kidney weight)^∗^ 100]

Animals were schedule 1 culled in compliance with United Kingdom Home Office regulations. Upon confirmation of death, blood was obtained via cardiac puncture and the animal perfused with PBS and tibia length recorded. For isolation of serum, blood was allowed to clot and spun down at 3000G for 20 min at 4°C. Organs were removed and weighed, prior to being cut into predefined sections with sections for RNA and protein snap frozen in liquid nitrogen, while those for histology were placed in 10% formalin for 24-h, before embedding in paraffin to produce FFPE sections. For flow cytometry studies kidney portions were collected in PBS on ice prior to processing.

### Histology

Three μM thick FFPE sections were cut and deparaffinized prior to staining with picrosirius red in accordance with manufacturer’s guidelines (Abcam, ab150681). Slides were imaged using ZEISS Axio Scan.Z1 Slide Scanner. Quantification of images was carried out using Image-Pro Premier 9.2.

### RNA Extraction, Gene and miRNA Expression

Tissue was homogenized using Qiagen TissueLyser II. RNA was extracted from homogenized tissue with the RNeasy Mini Kit (Qiagen 74106) and RNA yields were quantified using NanoDrop 1000 (Thermo Fisher). Reverse transcription was carried out using high-capacity cDNA synthesis kit (Applied Biosystems, 4368814). Quantitative real-time PCR (qRT-PCR) was carried out using specific Taqman gene probes (Table 1).

**TABLE 1:** Taqman gene and miRNA expression assays used for qRT-PCR in these studies.

**Gene**	**Probe/assay ID**
Col1a1	Mm00801666_g1
Col3a1	Mm01254476_m1
Col4a1	Mm01210125_m1
Acta2	Mm00725412_s1
Mmp2	Mm00439498_m1
Tgfb1	Mm01178820_m1
Il1b	Mm00434228_m1
Tnf	Mm00443258_m1
Ppia	Mm02342430_g1
Nppa	Mm01255747_g1
Nppb	Mm01255770_g1
Gapdh	Mm99999915_g1
miR-21-5p	000397
miR-214-3p	002306
u6	001973

### Renal Function Analysis

Timed overnight collections of urine (18 h) were performed at baseline, 6-weeks post-STNx and 10-weeks post-STNx from mice housed singly in metabolic cages. Blood was collected at baseline and at termination. Urine and serum were stored at −20°C prior to analysis by an in-house biochemical analysis service^2^.

Mouse urine albumin measurements were determined using a commercial Microalbumin Kit (DiaSys Diagnostics Systems, Germany) adapted for use on a Cobas Mira analyzer (Roche Diagnostics, Ltd., Welwyn Garden City, United Kingdom). The immunoturbidimetric assay was standardized against purified mouse albumin standards (Sigma Chemical, Co., Poole, United Kingdom) with samples diluted in phosphate buffer saline as appropriate. Within run precision was CV < 5% while intra-batch precision was CV < 7.1%.

Urine ion concentration was determined using ion-selective electrodes using the SPOTCHEM^TM^ E-Plate with the SPOTCHEM^TM^ EL Analyzer. Urine osmolality was measured by freezing-point depression on a Micro-Digital i-Osmometer (Type 16M, CamLab, United Kingdom).

### Echocardiography for Cardiac Structure and Function

Echocardiography was carried out by University of Edinburgh pre-clinical imaging facility under isoflurane anesthesia at 6 and 10-weeks post-surgery as previously published (Respress and Wehrens, 2010; Gao et al., 2011; Lindsey et al., 2018). A parasternal long-axis view of the heart was used to obtain EKV (ECG-gated Kilohertz Visualization) over one cardiac cycle. Spectral Doppler was carried out in parasternal short-axis view and used to assess mitral valve and blood-flow. Doppler sample volume was placed across the mitral valve for measurement of E (early) and A (late, atrial) wave velocity. Doppler sample volume was placed at mid-left ventricular level to measure isovolumic relaxation (IV RT).

### Blood-Pressure Analysis

Systolic blood-pressure was measured via a non-invasive tail-cuff method in a customized machine (Wang et al., 2017). Mice were trained prior to the start of the study. The mice were placed in a hot-box at 32°C for 5–10 min prior to blood-pressure measurement.

### Flow Cytometry

Tissue was placed in gentleMACS^TM^ C Tubes with digestion buffer (Collagenase Type II 0.425 mg/mL, Collagenase D 0.625 mg/mL, Dispase 1 mg/mL and DNAse 30 μg/mL) and dissociated using the gentleMACS^TM^ Dissociator. Cellular suspensions were digested at 37°C for 30 min then gentleMACS^TM^ dissociated for a second time. The cellular suspensions were then put through 100, 70, and 40 μM sieves sequentially and red blood lysis performed with Red Blood Cell Lysing Buffer (Sigma). The concentration of the resultant single cell suspension was determined using a cell counter and cells dispensed into 96-well round-bottom plate and incubated with appropriate rat anti-mouse antibodies (Table 2). Unstained samples, compensation beads for each antibody, FMO samples and cell suspensions were run on the six laser LSR Fortessa cell analyzer (BD Biosciences) using DAPI to determine live cells. Data was analyzed using FlowJo software.

**TABLE 2:** Antibodies utilized in flow cytometry.

**Antibody**	**Clone/flurochrome/final concentration**
Live	DAPI/1:1000
CD45	30-F11/APC or BV650/1:100
F4/80	BM8/Pe-Cy7/1:200
MHCII	M5/114.15.2/APC-Cy7/1:400
Ly6G	1A8/e450/1:200
Ly6C	HK1.4/AF700/1:200
CD11b	M1_70/PE Dazzle/1:1000
CD11c	N418/BV605/1:100
CD206	MR5D3/APC/1:200

### Statistical Analysis

A pilot study was performed using Col1a1 gene expression as the outcome measure. Power calculations derived from the pilot study determined that *n* = 9 mice in each group were required to ensure sufficient power (95%) to detect a 30% difference of in Col1a1 expression at a 5% level of significance. To account for mortality at 9% (combined anesthetic and model mortality), *n* = 10 mice/group was employed. For C57BL/6 study the results are from a pilot study performed on group size *n* = 6.

**FIGURE 1:**
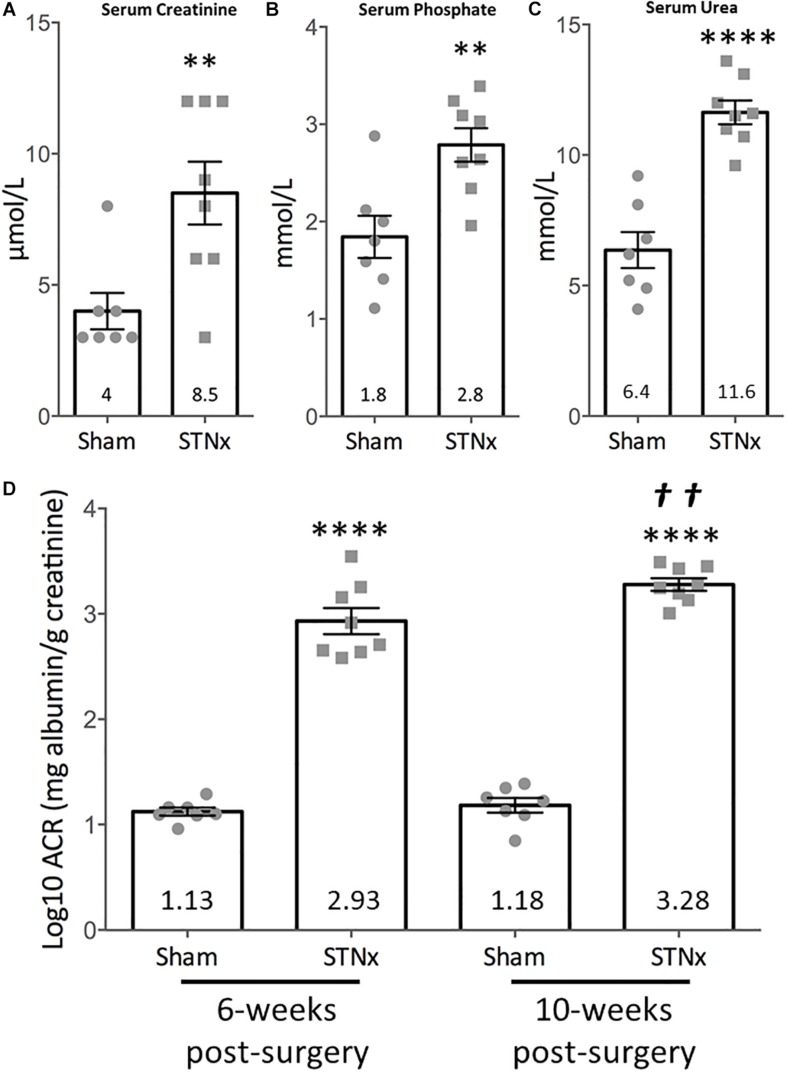
Effect of single-step subtotal nephrectomy on renal function. 129S2/SV mice were subjected to flank single-step STNx or sham surgery and were culled 10-weeks post-surgery. Urine and blood at 10-weeks post-STNx or sham surgery was analyzed for renal function parameters. **(A)** Serum creatinine, **(B)** phosphate, **(C)** urea. Sham: *n* = 7, STNx: *n* = 8. Student’s *t*-test was used for statistical analysis. ^∗∗^*P* ≤ 0.01, ^∗⁣∗⁣∗∗^*P* ≤ 0.0001. Plotted as mean ± SEM. **(D)** Urinary albumin:creatinine ratios (log10) were calculated from timed overnight (18 h) collections from animals 6 and 10-weeks post-surgery. Sham: *n* = 7, STNx: *n* = 8. All comparisons made via two-way ANOVA for repeat measures, with Sidak’s multiple comparisons test. ^∗⁣∗⁣∗∗^*P* ≤ 0.0001 vs. sham (of same timepoint), ^††^*P* ≤ 0.01 vs. STNx 6-week. Plotted as mean ± SEM.

**FIGURE 2:**
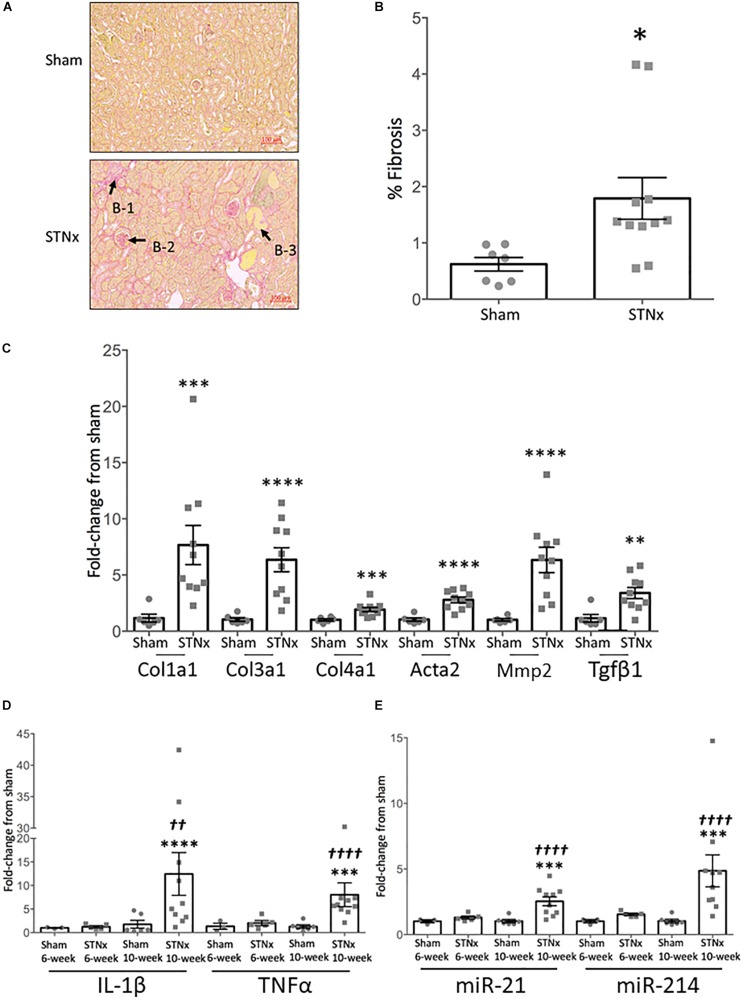
Single-step subtotal nephrectomy induces significant renal fibrosis. 129S2/SV mice were subjected to flank single-step STNx or sham surgery and were culled 10-weeks post-surgery. At sacrifice kidney sections were snap frozen for RNA analysis and to prepare FFPE 3 μM sections. **(A)** Picrosirius red staining for total collagen in kidneys. Exemplar images are provided at 5x magnification, scale bar 100 μM. B-1 = Tubulointerstitial fibrosis, B-2 = glomerulosclerosis, B-3 = tubular dilation. **(B)** Quantification of fibrosis (% PSR staining). Sham: *n* = 7, STNx: *n* = 11. Student’s *t*-test was used for statistical analysis. ^∗^*P* < 0.05. Plotted as mean ± SEM. **(C)** Pro-fibrotic gene expression in whole kidney tissue was determined by quantitative real-time PCR (qRT-PCR) carried out with specific Taqman probes for each gene, normalized to housekeeper Ppia. Sham: *n* = 6, STNx: *n* = 10. Col1a1 was found to have non-parametric distribution, therefore Mann–Whitney test was used, Student’s *t*-test was used for other genes. ^∗∗∗∗^*P* < 0.0001, ^∗∗∗^*P* < 0.001, ^∗∗^*P* < 0.01. Plotted as RQ mean ± SEM. **(D)** Inflammatory gene expression in whole kidney tissue at 6 and 10-weeks post-surgery was determined by quantitative real-time PCR (qRT-PCR) carried out with specific Taqman probes for each gene, normalized to housekeeper Ppia. Sham 6-week: *n* = 3, STNx 6-week: *n* = 5, Sham 10-week: *n* = 6, STNx 10-week: *n* = 10. All comparisons made via an ordinary two-way ANOVA with Tukey’s multiple comparisons test. ^∗∗∗^*P* < 0.001 vs. sham (of same timepoint), ^∗∗∗∗^*P* < 0.0001 vs. sham (of same timepoint), ^††^*P* < 0.01 vs. STNx 6-week, ^†⁣†⁣††^*P* < 0.0001 vs. STNx 6-week. Plotted as mean ± SEM. **(E)** Renal fibrosis-associated miRNA expression in whole kidney tissue at 6 and 10-weeks post-surgery was determined by quantitative real-time PCR (qRT-PCR) carried out with specific Taqman probes for each miRNA, normalized to housekeeper U6. Sham 6-week: *n* = 3, STNx 6-week: *n* = 5, Sham 10-week: *n* = 6, STNx 10-week: *n* = 10. All comparisons made via an ordinary two-way ANOVA with Tukey’s multiple comparisons test. ^∗∗∗^*P* < 0.001 vs. sham (of same timepoint), ^†⁣†⁣††^ *P* < 0.0001 vs. STNx 6-week. Plotted as mean ± SEM.

All data was assessed for normal distribution using the D’Agostino-Pearson normality test. Comparisons between two, normally-distributed, data points were carried out via Student’s *t*-test. Comparisons between two unpaired, non-normally distributed data points were carried out via Mann–Whitney test. All data generated was subjected to Grubbs outlier test, outliers were removed from analysis. ACR at 6 and 10-weeks post-surgery (Figure 1D) was assessed for statistical significance via two-way ANOVA for repeat measures with Sidak’s multiple comparison test. Gene and miRNA expression at 6 and 10-weeks post-surgery (Figures 2D,E) were compared via ordinary two-way ANOVA with Tukey’s multiple comparison test.

## Results

### Effect of One-Step Flank STNx on Renal Function

We utilized initially male 129S2/SV mice because they have been shown to be sensitive to developing renal dysfunction in previous studies. Following our refined single step flank subtotal nephrectomy (STNx) procedure there was no significant difference in body weight between the sham and STNx animals during the 10-week study run (Supplementary Figure 1A). The use of a single surgery procedure was well-tolerated. In total across four studies (pilot and full studies), 53 male 129S2/SV mice aged 7–10 weeks (weights 24.7 ± 0.37 g SEM) and 10 male Gli1 × Ai14 mice on a C57BL/6 genetic background aged 9 ± 3 weeks (weights 31.9 ± 1.1 g SEM) were subjected to STNx or sham surgery. There was an overall model failure rate of 9% (5% mortality, 2% anesthetic death, and 2% early termination rate due to deteriorating animal body condition scoring). Group housing the mice post-surgery resulted in improved animal condition scores and faster recovery compared to single housing (Supplementary Figure 1B). Animal stress peaked day 3 post-surgery as assessed by body condition score for single and grouped house, which may suggest analgesia up to day 2 post-surgery may be warranted (Supplementary Figure 1B). Across the studies the mean percentage of residual left kidney mass was 32.9 ± 0.98% SEM (Supplementary Figure 1C).

Initially we examined the effect of the STNx performed on 129S2/SV male mice on renal excretory function and proteinuria as patients with CKD have increased serum creatinine (Jha et al., 2013; Levey et al., 2014; Hill et al., 2016), urea (Jörres et al., 2004; Almeras and Argilés, 2009; Lau and Vaziri, 2016; Vanholder et al., 2018), and phosphate (Martin and González, 2011; Felsenfeld et al., 2015; Ritter and Slatopolsky, 2016; Vervloet et al., 2017) levels as well as proteinuria. Biochemical analysis of blood samples from mice 10-weeks post-STNx consistently revealed significant increases in serum creatinine (9.29 vs. 4 μmol/l) (Figure 1A), phosphate (2.74 vs. 1.84 mmol/L) (Figure 1B) and urea (11.62 vs. 6.35 mmol/L) (Figure 1C) compared to sham operated mice indicating a reduction of renal excretion. Total urinary albumin excretion was significantly increased 123-fold in STNx mice compared to controls (Table 3). STNx mice had a significant increase in urinary albumin:creatinine ratio (ACR) at 6 and 10-weeks post-surgery, with ACR significantly increasing from 6 to 10-weeks post-surgery indicating progressive proteinuria (Figure 1D). Renal sodium excretion was not significantly different between STNx and sham operated mice at both 6-weeks (174 ± 24 vs. 121 ± 26 μmol/18 h) and 10-weeks (167 ± 20 vs. 132 ± 24 μmol/18 h; Table 3). Chloride excretion was also not different between groups at 6-weeks (372 ± 38 vs. 290 ± 61 μmol/18 h) and 10-weeks (255 ± 21 vs. 271 ± 25 μmol/18 h; Table 3). Potassium excretion was significantly different between groups, reflecting an increase in excretion in STNX mice at 6-weeks (380 ± 32 vs. 260 ± 45 μmol/18 h, *P* = 0.019); potassium excretion was not different between groups at 10-weeks (234 ± 15 vs. 218 ± 28 μmol/18 h; Table 3). Urine osmolarity was also measured with no significant difference at 6-weeks between sham and STNx groups (1272.7 vs. 1157.5 mOsm) but by 10-weeks post-STNx there was significantly lower osmolarity compared with sham animals (Table 3).

**TABLE 3:** Urine parameters measured in male 129S2/SV mice 10-weeks post-STNx.

**Urine parameters**	**Sham (10-weeks)**	**STNx (10-weeks)**
Na excretion	131.94 ± 24 μM/18 h	166.77 ± 20 μM/18 h
K excretion	271.78 ± 28 μM/18 h	164.45 ± 21 μM/18 h
Cl excretion	270.84 ± 25 μM/18 h	225.75 ± 11 μM/18 h
Total albumin	3.39 ± 0.48 μg/18 h	418.16 ± 89 μg/18 h ^∗∗∗^
Osmolality	1347 ± 89 mOsm	914.5 ± 63 mOsm ^∗∗∗^

*Ion excretion, total albumin and osmolality was measured in 18 h-timed urine collection from mice 10-weeks post-surgery or age-matched sham mice. ^∗∗∗^*P* < 0.001 unpaired *t*-test.*

Renal fibrosis remains one of the best histological markers of progressive kidney disease (Ito et al., 2004; Hewitson, 2009; Hewitson et al., 2017). At 10-weeks post-STNx, renal fibrosis as measured by picrosirius red staining was increased 3.4-fold (increasing from 0.62 ± 0.12% in sham kidneys to 2.11 ± 0.37% in STNx kidneys) (Figures 2A,B). Evidence of tubulointerstitial fibrosis and glomerulosclerosis was observed along with tubular dilation (Figure 2A). 6-weeks post-STNx surgery, when ACR was already increased, there were no significant pro-fibrotic gene expression changes (Supplementary Figure 2). However, by 10-weeks post-STNx surgery, gene expression analysis revealed significant increases in the expression of pro-fibrotic genes (*Col1a1, Col3a1, Col4a1, Acta2, Mmp2, Tgf*β*1*) (Figure 2C). When gene expression of pro-inflammatory genes (*Il1*β and *Tnf*α) were examined there was no difference in expression compared to sham animals at 6 weeks but a significant increase from 6 to 10-weeks post-STNx (Figure 2D). We have previously reported that miR-21 and miR-214 are consistently elevated in the kidney following injury (Denby et al., 2011), however, these miRs have not been assessed in progressive renal dysfunction induced by STNx in 129S2/SV mice. We found that the pro-fibrotic miRNA miR-214-3p (Denby et al., 2014; Bai et al., 2019) was significantly upregulated 6-weeks post-STNx surgery (Figure 2E), prior to pro-fibrotic gene expression changes, with no change in the pro-fibrotic miRNA miR-21-5p expression (Denby et al., 2011; Chau et al., 2012; Gomez et al., 2015; Hennino et al., 2016). At 10-weeks post-STNx surgery, miR-21-5p was significantly upregulated 2.4-fold and miR-214-3p remained significantly elevated with a 3.5-fold higher expression compared to sham kidneys (Figure 2E). Significant increases in miR-214-3p and miR-21-5p expression were detected in the kidneys of STNx group animals between the 6 and 10-week post-surgery timepoints (Figure 2E).

### Effect of One-Step Flank STNx on Intra-Renal Inflammation

**FIGURE 3:**
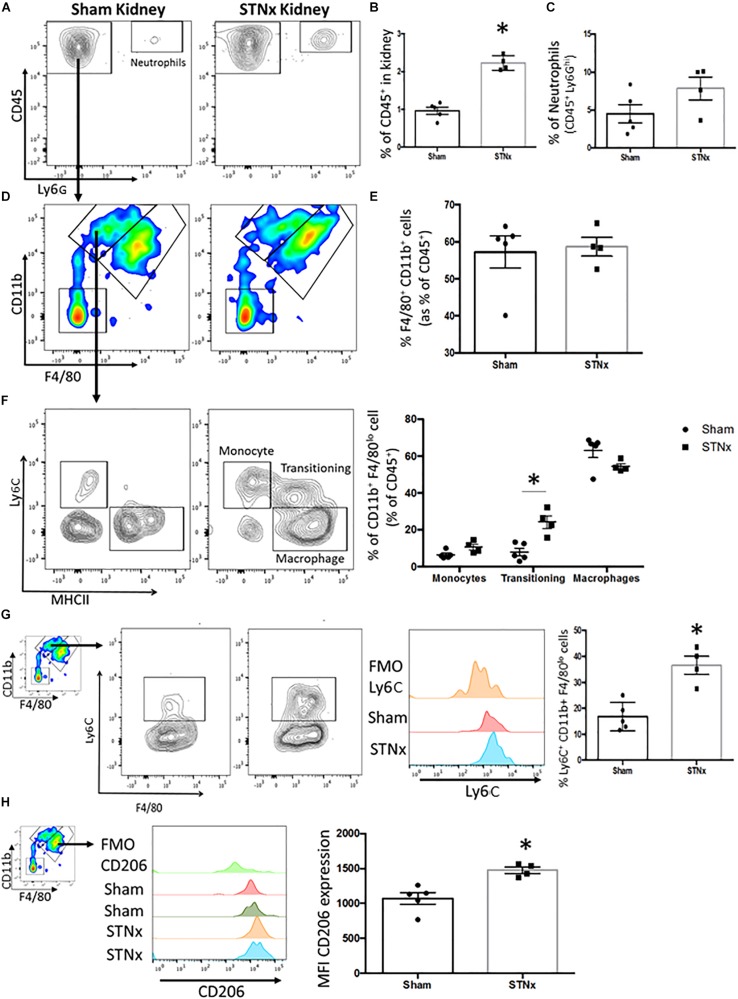
Analysis of inflammatory infiltrate in the STNx kidney. 129S2/SV mice were subjected to flank single-step STNx or sham surgery and were culled 10-weeks post-surgery. At sacrifice *n* = 5 Sham and *n* = 4 STNx kidneys were perfused and kidneys digested for flow cytometry and analyzed on the 6L Fortessa Flow Analyzer. **(A)** Plot of CD45^+^ cells and neutrophils (CD45 + Ly6G^*hi*^) in kidney. **(B)** Quantification of the percentage of total cells that express CD45^+^ in the kidney. **(C)** Quantification of Neutrophils (CD45^+^ Ly6G^*hi*^) in the kidney. **(D)** Analysis of CD45^+^ Ly6G^–^ CD11b^+^ F4/80^+^ population in the kidney. **(E)** Quantification of CD45^+^ Ly6G^–^ CD11b^+^ F4/80^+^ population in the kidney. **(F)** Subset analysis and quantification of CD45^+^ Ly6G^–^ CD11b^+^ F4/80^*lo*^ population into monocytes (Ly6C^*hi*^ MHCII^–^), transitioning monocyte-macrophages and macrophages (Ly6C^–^ MHCII^+^). **(G)** Expression and quantification of the proportion of CD45^+^ Ly6G^–^ CD11b^+^ F4/80^*lo*^ that express Ly6C. **(H)** Analysis and quantification of CD206 expression in the resident macrophage population CD45^+^ Ly6G^–^ CD11b^+^ F4/80^*hi*^. *N* = 5 Sham *N* = 4 STNx statistical analysis by Mann–Whitney test ^∗^*P* < 0.05.

As we observed an increase in pro-inflammatory gene expression markers 10-weeks post-STNx, we sought to further characterize the nature of the inflammatory cells in kidneys from mice that underwent STNx. Analysis by flow cytometry (gating strategy provided in Supplementary Figure 3A) revealed that at 10-weeks post-STNx there was a significant increase in the proportion of cells in the kidney that expressed CD45^+^ compared to sham kidneys (2.23% STNx vs. 0.96% Sham, Figures 3A,B and Supplementary Figure 3B). Similarly, a significant increase in CD45^+^ cells was also observed in the hearts of animals subjected to STNx (Supplementary Figure 3C). Further analysis of the CD45^+^ population in the kidney revealed no significant difference in the proportion of CD45^+^ inflammatory cells constituted by neutrophils (7.9% STNx vs. 4.5% Sham; Figure 3C), CD11b^+^ F4/80^*lo*^ macrophages (monocyte derived), or CD11b^+^ F4/80^*hi*^ macrophages (resident population) (Figures 3D,E). However, further subset analysis of the CD11b^+^ F4/80^*lo*^ population revealed a clear waterfall effect in the STNx kidneys with Ly6C^*hi*^ monocytes transitioning to Ly6C^*lo*^ MHCII^+^ macrophages, an effect which was absent in the sham kidney (Figure 3F). Furthermore, the percentage of CD45^+^ CD11b^+^ F4/80^*lo*^ Ly6C^*hi*^ cells was significantly increased in the STNx kidneys (Figure 3G). Analysis of the CD11b^+^ F4/80^*hi*^ resident macrophages population revealed that there was significantly increased expression of CD206 in these resident macrophages in the STNx kidneys (Figure 3H).

### Effect of One-Step Flank STNx on Vascular and Cardiac Parameters

**FIGURE 4:**
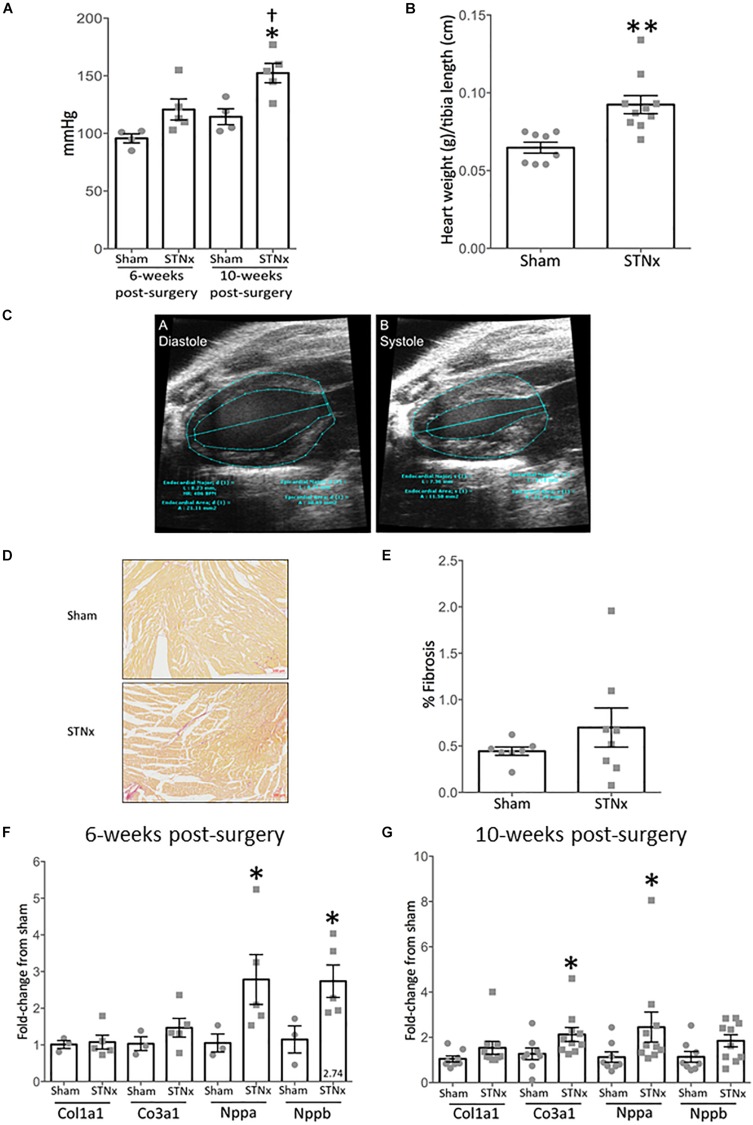
Single-step flank STNx induces cardiac dysfunction and hypertrophy but not cardiac fibrosis. 129S2/SV mice were subjected to flank single-step STNx or sham surgery and were culled 10-weeks post-surgery. At sacrifice heart sections were taken and snap frozen for RNA analysis and to prepare FFPE 3 μM sections. **(A)** Systolic blood pressure was measured via tail cuff at 6 and 10-weeks post-surgery. Sham: *n* = 4, STNx: *n* = 5. One way-ANOVA with Tukey’s multiple comparison test, all compared to sham. ^∗^*P* ≤ 0.05, ^†^*P* < 0.05 vs. STNx at 6 weeks. Plotted as mean ± SEM. **(B)** Heart weight at cull normalized to tibia length. Sham: *n* = 7, STNx: *n* = 11. Student’s *t*-test was used for statistical analysis. ^∗∗^*P* ≤ 0.01. Plotted as mean ± SEM. **(C)** Exemplar images of the analysis of EKV echocardiography via VisualSonics software. A = diastole, B = systole. Trace lines were drawn along the epicardial and endocardial borders at both end systole and end diastole. Left ventricle (LV) major axes were also traced at end systole and end diastole by drawing a line from the LV apex endocardium or LV apex epicardium, to the mitral valve line. **(D)** Picrosirius red stain for total collagen in hearts. Exemplar images are provided at 5x magnification. **(E)** Quantification of fibrosis (% PSR staining) carried out via Image-Pro Plus 7. Sham: *n* = 7, STNx: *n* = 8. Student’s *t*-test was used for statistical analysis. Plotted as mean ± SEM. **(F)** Gene expression in whole heart tissue was assessed 6 weeks post-STNx using quantitative real-time PCR (qRT-PCR) using specific Taqman probes for each gene, normalized to housekeeper Gapdh. Sham: *n* = 3, STNx: *n* = 5. Student’s *t*-test was used for statistical analysis. ^∗^*P* ≤ 0.05. Plotted as mean ± SEM. **(G)** Gene expression in whole heart tissue 10-weeks post-STNx or sham surgery was assessed using quantitative real-time PCR (qRT-PCR) using specific Taqman probes for each gene, normalized to housekeeper Gapdh. Sham: *n* = 8, STNx: *n* = 10. Col1a1, Col3a1, and Nppa expression were determined to have non-parametric distribution. Mann–Whitney test was used for statistical analysis on Col1a1, Col3a1 and Nppa, Student’s *t*-test was used on Nppb. ^∗^*P* ≤ 0.05. Plotted as mean ± SEM.

We next determined the effect of the STNx surgery on vascular and cardiac function in the 129S2/SV mice we had measured renal excretory function and proteinuria. We determined systolic blood pressure using tail vein plethysmograph in trained conscious mice at baseline, at 6-weeks post-surgery and at study end. The mean systolic blood pressure at 6-weeks was not significantly different in STNx mice compared with sham animals, but was significantly increased from 115 ± 2.6 mmHg in sham animals to 153.1 ± 4.6 mmHg in STNx animals 10-weeks post-STNx surgery (Figure 4A). The STNx mice also had significantly increased heart mass compared to sham animals at 10-weeks post-STNx surgery (Figure 4B). Therefore, we sought to determine the effects of the progressive loss of renal function induced by our one-step STNx surgery in the 129S2/SV mice on cardiac function as measured by echocardiography (ECHO) carried out at baseline, 6- and 10-weeks post-surgery (Figure 4C and Table 4). No significant differences in percentage ejection fraction were detected, although there was a trend for a reduction 10-weeks post-STNx. At 6-weeks, changes were detected in % fractional area change (FAC) and area change, indicating that adaption had begun to occur at this point (Table 4), but no statistical difference was detected in heart weight (data not shown). By 10-weeks, STNx animals had increased cardiac wall thickness (0.88 ± 0.02 to 1.05 ± 0.04 mm) and left-ventricle mass (182.7 ± 9.06 to 234.6 ± 17.75 mm) compared with sham animals (Table 4), which mirrored the increased heart weights measured at 10-weeks post-STNx (Figure 4B). Doppler imaging performed on the mitral valve revealed a significantly increased left ventricle isovolumetric relaxation time (IV RT) at 10-weeks post-STNx, but not 6-weeks post-STNx, suggesting STNx induced renal dysfunction may lead to diastolic dysfunction over time (Lindsey et al., 2018; Schnelle et al., 2018). As ECHO analysis suggested cardiac hypertrophy and diastolic dysfunction had occurred, cardiac fibrosis was assessed histologically. At 10-weeks post-STNx there was no significant increase in total collagen deposition in the heart (Figures 4D,E). At 6-weeks post-STNx, no change in expression for fibrillar collagen genes *Col1a1* and *Col3a1* was observed (Figure 4F), however *Col3a1* expression was significantly increased at 10-weeks compared with sham animals (Figure 4G). There was significantly higher expression of the cardiac hypertrophy markers ANP (Nppa) and BNP (Nppb) in the STNx compared with sham animals at 6-weeks with ANP remaining increased a 10-weeks (Figures 4F,G).

**TABLE 4:** Cardiac echocardiography measurements in STNX and Sham animals at 6 and 10-weeks post-surgery.

	**6-weeks post-surgery**		**10-weeks post-surgery**	
	**Sham**	**STNx**	**Sham**	**STNx**
EKV:	*n* = 8	*n* = 10	*n* = 8	*n* = 10
Ejection fraction (%)	59.1 ± 1.5	48.8 ± 4.4	54.4 ± 4	45.9 ± 2.8
Fractional area change (%)	40.5 ± 0.8	32.2 ± 3.3 **^∗^**	37.2 ± 3.2	29.8 ± 2.3
Area change (mm^2^)	9.21 ± 0.8	7.21 ± 0.64 **^∗^**	8.73 ± 0.86	6.91 ± 0.34 ^†^
Cardiac wall thickness (mm)	0.92 ± 0.03	1.02 ± 0.04	0.88 ± 0.02	1.04 ± 0.04 ^††^
LV mass (mg)	189.1 ± 10.7	215.8 ± 16.3	182.7 ± 9.1	234.6 ± 17.7 ^†^
Mitral valve spectral doppler:	*n* = 8	*n* = 9	*n* = 8	*n* = 9
E wave (mm/s)	770 ± 27.4	650 ± 41.7**^∗^**	747 ± 25.6	663 ± 38.9
A wave (mm/s)	457 ± 24.5	479 ± 44.3	443 ± 39.9	471 ± 45.5
LV IV RT	24.5 ± 1.24	28.2 ± 1.75	24.1 ± 0.82	28.6 ± 1.38 ^†^

*Echocardiography measurements were obtained under isoflurane anesthesia at 6 and 10-week post-STNx or sham surgery. Sham: *n* = 8, STNx: *n* = 10. Student’s *t*-test was used for statistical analysis of difference between sham and STNx groups at 6 or 10-weeks post-surgery. ^∗^*P* ≤ 0.05 at 6-weeks vs. sham. ^†^*P* ≤ 0.05, ^††^*P* ≤ 0.01 at 10-weeks vs. sham.*

**FIGURE 5:**
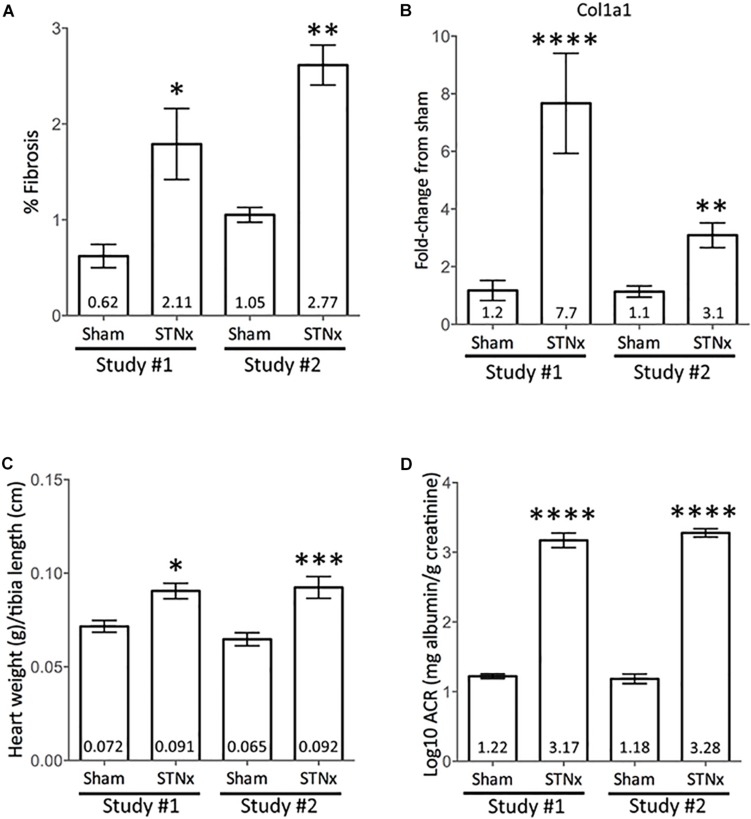
Comparison between independent STNx studies conducted in male 129S2/SV mice. Male 129S2/SV mice (bought from Envigo) were subjected to STNx in two separate studies, 1.5 years apart (Study 1 – March 2017; Study 2 – September 2018). Plotted as mean ± SEM. **(A)** Quantification of picrosirius red stain for total renal collagen, via Image-Pro Plus. Study #1 Sham: *n* = 7, study #1 STNx: *n* = 11, Study #2 Sham: *n* = 7, study #2 STNx: *n* = 6. **(B)** Renal Col1a1 gene expression at 10-weeks post-surgery. Study #1 Sham: *n* = 6, study #1 STNx: *n* = 10, Study #2 Sham: *n* = 8, study #2 STNx: *n* = 10. **(C)** Heart weight normalized to tibia length at cull, 10-weeks post-surgery. Study #1 Sham: *n* = 7, study #1 STNx: *n* = 11, Study #2 Sham: *n* = 8, study #2 STNx: *n* = 10. **(D)** Log10 albumin:creatinine ratio (ACR) at 10-weeks post-surgery. Study #1 Sham: *n* = 7, Study #1 STNx: *n* = 11, Study #2 Sham: *n* = 7, study #2 STNx: *n* = 8. Statistical analysis carried out via ordinary one-way ANOVA with Tukey’s multiple comparisons test. ^∗^*P* < 0.05 vs. sham (same study), ^∗∗^*P* < 0.01, ^∗∗∗^*P* < 0.001, ^∗∗∗∗^*P* < 0.0001.

To demonstrate consistent outputs from the STNx model presented in this manuscript, two independent studies were compared in male 129S2/SV mice run 1.5 years apart (Figure 5). Between the two studies there were no significant differences in the amount of renal fibrosis detected, the increase in ACRs induced, amount of LVH (measured by heart weight:normalized to tibia) or gene expression of collagen 1 in renal tissue (Figure 5).

### Effect of Genetic Background on One-Step Flank STNx Induced Renal and Cardiac Dysfunction

Subtotal nephrectomy is known to be a strain-dependent model, with C57BL/6 mice being resistant to developing fibrosis (Ma and Fogo, 2003; Leelahavanichkul et al., 2010). However, this strain is commonly used as a background for the production of transgenic animals. To assess how the single step STNx performs in this genetic background, a Gli1 reporter mouse (Gli1 × Ai14) on a C57BL/6 background was used. An increase in total collagen expression (sham: 0.81 ± 0.03%, STNx: 2.57 ± 0.42%) along with histological hallmarks of renal fibrosis (tubulointerstitial fibrosis and glomerulosclerosis) was observed (Supplementary Figures 4A,B), as well as a significant increase in pro-fibrotic genes and inflammatory genes (Supplementary Figure 4C). However, the urinary ACRs of STNx animals were not different to sham at 6 or 10-weeks post-surgery (Supplementary Figure 4D) and there was no increase in heart weight observed (Supplementary Figure 4E).

## Discussion

Clinically, CKD is characterized by worsening excretory function with or without proteinuria and renal biopsies typically show glomerulosclerosis, tubulointerstitial fibrosis, and inflammatory cell infiltrates. In addition, CKD is associated with the development of CVD, characterized by hypertension and cardiac and vascular dysfunction. Therefore, we set out to establish if a one-step flank STNx performed in male 129S2/SV mice could model these clinical CKD parameters in a consistent and robust manner.

Urinary albumin to creatinine ratio (ACR) is an important prognostic indicator for progression of renal disease (Feldman et al., 2014; Vassalotti et al., 2016), and cardiovascular events (Waheed et al., 2012). Furthermore, regression of albuminuria is associated with improved renal outcomes (Perkins et al., 2007) and hence represents a therapeutic target in CKD patients (Heerspink and Gansevoort, 2015). Hence, the significantly increased ACR in STNx mice compared to sham operated mice represents an important clinically-relevant readout for testing novel therapeutics in this model. Indeed, this was a key readout used to demonstrate the efficacy of ACE inhibition in seminal studies in the rat subtotal nephrectomy model (Meyer et al., 1985; Anderson et al., 1986), which represents one of the few therapies that have been successfully translated from rodent models to human CKD. Urinary excretion of sodium, chloride and potassium were largely comparable between sham-operated and STNx mice at both time points. Although GFR was not directly measured here, the major reduction in filtration following STNx would significantly reduce the filtered load for sodium, chloride and potassium and our data indicate a proportionate decline tubular reabsorption. Thus, over this time course, the renal tubule adapted to maintain electrolyte excretion and preserve balance. The STNx group showed a progressive decline in urine osmolarity, indicating a reduced concentrating capacity in the remnant kidney.

Male 129S2/SV mice subjected to the refined one-step STNx developed increased serum creatinine, BUN and phosphate which are important clinical manifestations of renal disease. There was approximately a twofold increase in both creatinine and urea by 10-weeks post-STNx, however, the coefficient of variation (CV) for urea was only 11.1% compared to 39.8% for creatinine suggesting that serum urea measurements are more reliable as kidney injury markers in rodents. In this context, it would be interesting to measure some newer markers of renal function such as Cystatin C (Song et al., 2009) to further validate this model of progressive renal dysfunction in mice. The increase in serum phosphate is important as hyperphosphatemia is observed in late-stage CKD and is a driver of secondary hyperparathyroidism (Locatelli et al., 2002), mineral bone disorder and vascular calcification (Felsenfeld et al., 2015). Hyperphosphatemia is also thought to contribute to cardiac hypertrophy and calcification of heart valves and conduction system (Di et al., 2015).

The serum urea levels recorded in the mice following the single step flank STNx procedure were significantly increased compared to age matched sham controls, however, these levels are lower than those observed in some other studies which might suggest this surgery resulted in less tissue being taken and a less severe renal dysfunction being induced which resulted in less mortality. The lack of a statistical difference in body weight appears to support this too. There are very few studies that document the amount of tissue taken beyond those studies that showed the relationship between renal function and the amount of tissue removed (Rambausek et al., 1985). In our studies in male SV129/SV mice, when on average 32.9% of left renal tissue remained, all mice developed renal dysfunction with significant alterations in renal excretory function and proteinuria. Importantly, the low mortality rate in this STNx model means animals survived to pre-defined study end-points which maintained statistical power in the studies.

The relationship between CKD and hypertension is bidirectional. CKD is known to cause hypertension and hypertension is a known risk factor for CKD (Gosmanova and Kovesdy, 2016). 86% of CKD patients have hypertension (Gosmanova and Kovesdy, 2016). Blood pressure lowering strategies have been shown to decrease progression of kidney disease and all-cause mortality in patients with CKD (Peters et al., 2017). At 10-weeks post-STNx, a significant increase in systolic blood pressure was detected using tail vein plethysmograph. This was in agreement with several other studies (Kennedy et al., 2008; Leelahavanichkul et al., 2010; Gava et al., 2012). However, this is contrast to subtotal nephrectomy two-step models run in 129SV and FVB mice, where no change in blood pressure was observed (Siedlecki et al., 2009; Dilauro et al., 2010). Interestingly, sham mice from the 129SV study had higher systolic blood pressures than recorded in our study (129 ± 4 mmHg; Siedlecki et al., 2009 sham versus 115 ± 2.6 mmHg sham operated in our study). The mice in this study were trained prior to blood pressure measurements to ensure the results were not affected by stress of the procedure, potentially explaining the lower conscious blood pressures obtained. However, the diet of the mice differing between studies could also explain these effects as increased dietary NaCl is known to aggravate hypertension. Similarly, group housing of the animals may also have affected our results, as this is a less stressful environment for the animals that may have led to our sham animals having lower systolic blood pressure.

The refined single-step flank STNx performed on 129S2/SV mice results in significant renal fibrosis, accompanied by histological hallmarks tubulointerstitial fibrosis (TIF) and glomerulosclerosis (GS) by 10-weeks post-surgery. Both TIF and GS are observed in CKD patients (Nakagawa et al., 2015), with TIF being an important predictor of disease progression (Nath, 1992). Increases in three collagen genes mirror what can be seen histologically. *Col1a1* and *Col3a1* encode fibrillar collagens (Delella et al., 2017) which are important extracellular matrix components (Nakagawa and Duffield, 2013). *Col4a1* is an important basement membrane component (Jones et al., 2016), suggesting possible basement membrane expansion in STNx kidneys. *Acta2* encodes α-smooth muscle actin, a marker of activated myofibroblasts, which increase in number in renal fibrosis (Bernard et al., 2014) and secrete excess extracellular matrix components (Bernard et al., 2014). The increased expression of miR-21 and miR-214 is of interest given the pro-fibrotic role these miRNAs are known to have in the kidney (Denby et al., 2014). Importantly, miR-21 and miR-214 have also been shown to be up-regulated in the kidneys of patients with CKD, indicating that the STNx model mimics the mechanisms that promote fibrosis in human disease (Lv et al., 2018). Taken together, increased expression of these genes and miRNAs in the kidney suggests a pro-fibrotic environment is present in the kidneys of mice subjected to STNx on both the histological and molecular level.

Gene expression of the cardiac hypertrophy marker ANP (Kerkelä et al., 2015; Riaz et al., 2015) was increased in hearts at both 6 and 10-weeks post-STNx whilst BNP (Kerkelä et al., 2015) was only significantly increased at 6-weeks. This coupled with the increased heart weight detected at 10-weeks post-STNx and increased average cardiac wall thickness and left-ventricular mass detected by ECHO at 10-weeks post-STNx suggests significant hypertrophy had taken place, although cardiac fibrosis had not manifested at the histological level at this time-point. However, no significant change in these measures was detected at 6-weeks post-STNx, indicating the development of this cardiac hypertrophy in the STNx model is time dependent. Prolonged isovolumic relaxation time (IV RT) was observed at 10-weeks post-STNx (but not at 6-weeks), indicating diastolic dysfunction is present in these mice via impairment of myocardial relaxation (Schnelle et al., 2018). No significant decrease in ejection fraction was observed, indicating that adaptive measures taken by the heart to overcome the increased stress have been successful up until 10-weeks post-STNx. It is likely necessary to extend the timeline of the model past 10-weeks post-surgery in order to observe diastolic dysfunction and fibrosis.

The STNx model has been extensively reviewed from the point of heart-kidney interactions (Bongartz et al., 2012; Hewitson et al., 2015; Liu, 2019). A common comment in reviews of the literature is that in mice this model is highly variable if not uniformly performed and you can get conflicting results depending on the strain. Here we are able to present data which shows that in 129S2/SV male mice you can achieve reproducible physiological readouts using the single step STNx model described, with the added advantage of low mortality and improved animal welfare. The model is amenable to echocardiography studies which allow longitudinal studies of cardiac structure and function. Utilizing the 129S2/SV mouse also allows for the inflammatory cell profile to be examined in detail as multiple validated antibodies are available for mouse unlike rat.

Low grade inflammation is common in CKD patients (Amdur et al., 2016), with patients typically exhibiting elevated CRP, TNFα, and IL-6 levels (Panichi et al., 2001; Lacson and Levin, 2004). Furthermore, macrophage infiltration into the kidney in CKD has been found to correlate with a decline in kidney function (Eardley et al., 2006; Amdur et al., 2016). The analysis of the inflammatory cell content of the STNx mice revealed there is increased CD45^+^ hematopoietic cell content in the STNx kidney and heart. The STNx model mimics the findings in human CKD of increased inflammation, with flow cytometry data demonstrating persistent Ly6C^*hi*^ monocyte recruitment to the STNx kidney where they transition into pro-inflammatory macrophages. Previous work has identified a CD11b^+^ Ly6C^*hi*^ population to be induced with the onset of renal injury following ischemia reperfusion injury and unilateral ureteric obstruction (Lin et al., 2009; Clements et al., 2016). Importantly, in our study we excluded Ly6G^+^ neutrophils and found that significantly more CD45^+^ Ly6G^–^ CD11b^+^ F4/80^*lo*^ cells express Ly6C. This population has been previously shown to have a pro-inflammatory gene signature (Clements et al., 2016), and promote fibrosis in other organs such as the liver (Ramachandran et al., 2012). Within the CD45^+^ CD11b^+^ F4/80^*hi*^ resident macrophage population there was a significantly greater expression of CD206 (Mannose receptor 1, Mrc1). This C-type lectin is expressed predominantly by tissue macrophages and is involved in phagocytosis and acts as a scavenger receptor (Taylor et al., 2005). CD206 is classically thought of as an alternatively activated or M2 macrophage marker (Murray et al., 2014). The increase in the Mrc1 expression is confined to the resident macrophage population that may suggest that resident macrophages may play an important role in scavenging of debris and scar tissue. Hence, this STNx model in mice affords an opportunity in future studies to perform detailed mechanistic studies of the role of each immune cell subset in progressive CKD.

A number of refinements to traditional methods of undertaking subtotal nephrectomy were employed in the refined single-step STNx surgery used in this study with a key focus on animal welfare. One of the key differences between the surgical methods presented in this paper and the majority of previously published studies is the use of a single-surgery via flank incisions to perform both the nephrectomy and contralateral partial nephrectomy. Multiple papers have been published where nephrectomy is performed in one surgery, then 1–2 weeks later, resection of the poles or renal artery ligation is performed in a separate surgery (Kren and Hostetter, 1999; Ma and Fogo, 2003; Soler et al., 2008; Windt et al., 2008; Leelahavanichkul et al., 2010; Yang et al., 2010; Babelova et al., 2012; Gava et al., 2012; Li et al., 2012; Purnomo et al., 2013; Hyde et al., 2014; Ucero et al., 2014; Vavrinec et al., 2016; Rosendahl et al., 2018). Reducing the number of surgeries to which the mice are subjected, by performing the single-step STNx surgery has a number of benefits for animal welfare including: the animals undergo anesthesia on one less occasion, the length of time the mice are on-procedure is decreased, and requirement for analgesics is reduced. Our approach also avoids hypertrophy of the remaining kidney or remnant kidney (depending on the surgical order) between surgeries. Renal tissue during this phase could be argued to be in the “regenerative mode” and thus may be more resistant to the development of fibrosis which would not be present in this refined STNx model. Animal welfare in response to STNx was monitored weekly throughout the study using an animal condition scoring sheet (Supplementary Table 1), which included body condition scoring. A cumulative score of 5 or higher resulted in a mandatory schedule 1 termination of the animal. We found that group housing resulted in improved animal welfare scores, therefore group housing animals is recommended. Decreased weight and body condition score in mice is an important determinant of health status (Foltz and Ullman-Culleré, 1999; Ullman-Culleré and Foltz, 1999; Burkholder et al., 2012) and mice subjected to STNx were found not to differ in weight in comparison to sham animals at any time-point during the 10-weeks between surgery and sacrifice. In studies which use traditional two-step subtotal nephrectomy protocols, most (Leelahavanichkul et al., 2010; Gava et al., 2012; Zeng et al., 2018) but not all (Siedlecki et al., 2009), report a reduction in body weight with subtotal nephrectomized animals compared to sham controls. The lack of a difference in body weight may be due to this model inducing milder progressive renal disease as systolic blood pressure and ACRs were not significantly increased until 6-weeks post-surgery. However, this may also be in part due to improved animal welfare brought about by group housing. This study could have benefited from a side by side comparison with the traditional two-step STNx to fully demonstrate its advantages, however, the mortality rates for this surgery can be high and in a recent study shown to be 60% 4 weeks post-surgery (Tan et al., 2019). Therefore, in the interests of animal welfare it is not appropriate to run such a study.

The mortality following subtotal nephrectomy has been poorly reported in the literature, but is often high, for example 43% by 12-weeks post-surgery (Ma and Fogo, 2003). Other studies report no mortality but it is not clear if this included animals that either did not develop significant renal dysfunction or were terminated prematurely due to animal welfare concerns. In our model in total 9% of animals did not complete the study due to a combination of mortality during follow-up (*n* = 3), exceeding animal welfare scoring limits (*n* = 2) or failure to recover from anesthetic (*n* = 2). These data suggest that the single-step STNx surgery is well-tolerated by the mice, although a small mortality rate needs to be factored into power calculations.

The subtotal nephrectomy is known to be a strain-dependent model. Both the 129S2/SV and CD1 strain have been reported to be permissive to injury (Ma and Fogo, 2003; Kennedy et al., 2008; Siedlecki et al., 2009; Leelahavanichkul et al., 2010). In our 129S2/SV mice no cardiac fibrosis was detected *per se* but on the gene expression level Collagen III was increased by 10-weeks post-surgery. Using the CD1 strain of mice, cardiac fibrosis can be induced with the added insult of additional dietary salt (Fontes et al., 2015) which may be required in this model too. In a pilot study in this genetic background, the refined STNx model resulted in significant renal fibrosis and increased pro-fibrotic gene expression in the kidney which matches that previously observed (Ma and Fogo, 2003). However, there were no functional alterations detectable, e.g., increased ACR or changes in heart weight. This indicates that STNx in C57BL/6 mice may not be the best pre-clinical model to test novel therapies where clinically relevant renal and cardiac outcomes are required. These results mirror the experience of other groups with subtotal nephrectomy in C57BL/6 mice (Ma and Fogo, 2003; Leelahavanichkul et al., 2010), with additional stimuli such as angiotensin II infusion required to produce hard renal outcomes (Leelahavanichkul et al., 2010). The resistance to development of albuminuria in the C57BL/6 mice is well-recognized, albumin overloaded 129S2/SV mice develop abundant albuminuria whereas C57BL/6 show none despite increased serum albumin (Ishola et al., 2006). The significant increase in fibrosis observed, however, suggests that for studies into renal fibrosis, the STNx model could be further utilized to understand the precise pathophysiology of progressive renal fibrosis using genetic knockout mice on the C57BL/6 genetic background.

### Summary

Together, these data provide evidence that conducting the subtotal nephrectomy model with our refined protocol in male 129S2/SV mice results in renal dysfunction, renal inflammation, and fibrosis with systemic pathologies akin to what is observed in patients.

This model is also suitable for testing new therapies for CKD given its progressive nature, clinically relevant biochemical measurements and cardiac dysfunction. In addition, these therapies can be given with standard therapy of ACEi to examine physiological effects beyond those offered by blood pressure reduction alone.

C57BL/6 mice as previously reported are refractory to proteinuric renal dysfunction, blood pressure and cardiac changes but do develop significant renal fibrotic disease. Therefore, for pathophysiological studies of fibrosis the STNx model in C57BL/6 background may offer some insight when using genetic knockout models on this genetic background.

## Data Availability Statement

All datasets generated for this study are included in the article/Supplementary Material.

## Ethics Statement

The animal study was reviewed and approved by University of Edinburgh Animal Welfare and Ethical Review Board.

## Author Contributions

JO’S contributed to the investigation, analysis, the writing and revising of the original draft of the manuscript. SF contributed to the investigation, analysis, the writing and editing of the original draft of the manuscript. OT and AB contributed to the investigation and analysis. CC contributed to the investigation, analysis, and the editing of the original draft of the manuscript. MB contributed to the analysis and the editing of revised manuscript. AT contributed to the investigation, methodology, and analysis of the study. JH contributed to the conceptualization and methodology of the study. CB contributed to the methodology, analysis, and the writing, the review and the editing of the manuscript. BC contributed to the conceptualization, methodology, analysis, and the writing, the review and the editing of the manuscript. LD contributed to the funding acquisition, supervision, conceptualization, project administration, methodology, investigation, analysis, the writing, the review and the editing original, and revised draft of the manuscript.

## Conflict of Interest

LD is an awardee and JO’S is a recipient of an MRS Ph.D. studentship co-funded by Regulus Therapeutics. Regulus Therapeutics had no input on the studies contained in this manuscript. The remaining authors declare that the research was conducted in the absence of any commercial or financial relationships that could be construed as a potential conflict of interest.

## Footnotes

1 http://random.org

2 https://surf.ed.ac.uk/facilities/specialist-assay-service/

## References

[B1] Alicic R. Z. Rooney M. T. Tuttle K. R. (2017). Diabetic kidney disease: challenges, progress, and possibilities. *Clin. J. Am. Soc. Nephrol.* 12 2032–2045. 10.2215/CJN.11491116 28522654 PMC5718284

[B2] Almeras C. Argilés À (2009). The general picture of uremia. *Semin. Dial.* 22 329–333. 10.1111/j.1525-139X.2009.00575.x 19708976

[B3] Amdur R. L. Feldman H. I. Gupta J. Yang W. Kanetsky P. Shlipak M. (2016). Inflammation and progression of CKD: the CRIC study. *Clin. J. Am. Soc. Nephrol.* 11 1546–1556. 10.2215/CJN.13121215 27340285 PMC5012490

[B4] Anderson S. Rennke H. G. Brenner B. M. (1986). Therapeutic advantage of converting enzyme inhibitors in arresting progressive renal disease associated with systemic hypertension in the rat. *J. Clin. Invest.* 77 1993–2000. 10.1172/jci112528 3011863 PMC370560

[B5] Babelova A. Avaniadi D. Jung O. Fork C. Beckmann J. Kosowski J. (2012). Role of Nox4 in murine models of kidney disease. *Free Radic. Biol. Med.* 53 842–853. 10.1016/j.freeradbiomed.2012.06.027 22749956

[B6] Bai M. Chen H. Ding D. Song R. Lin J. Zhang Y. Y. (2019). MicroRNA-214 promotes chronic kidney disease by disrupting mitochondrial oxidative phosphorylation. *Kidney Int.* 95 1–16. 10.1016/j.kint.2018.12.028 30955870

[B7] Bain C. C. Hawley C. A. Garner H. Scott C. L. Schridde A. Steers N. J. (2016). Long-lived self-renewing bone marrow-derived macrophages displace embryo-derived cells to inhabit adult serous cavities. *Nat. Commun.* 7:ncomms11852. 10.1038/ncomms11852 27292029 PMC4910019

[B8] Bernard M. Dieude M. Yang B. Hamelin K. Underwood K. Hebert M.-J. (2014). Autophagy fosters myofibroblast differentiation through MTORC2 activation and downstream upregulation of CTGF. *Autophagy* 10 2193–2207. 10.4161/15548627.2014.981786 25495560 PMC4502773

[B9] Bongartz L. G. Braam B. Gaillard C. A. Cramer M. J. Goldschmeding R. Verhaar M. C. (2012). Target organ cross talk in cardiorenal syndrome: animal models. *Am. J. Physiol. Ren. Physiol.* 303 1253–1263. 10.1152/ajprenal.00392.2012 22914779

[B10] Boor P. Ostendorf T. Floege J. (2010). Renal fibrosis: novel insights into mechanisms and therapeutic targets. *Nat. Rev. Nephrol.* 6 643–656. 10.1038/nrneph.2010.120 20838416

[B11] Burkholder T. Foltz C. Karlsson E. Linton C. G. Smith J. M. (2012). Health evaluation of experimental laboratory mice. *Curr. Protoc. Mouse Biol.* 2 145–165. 10.1002/9780470942390.mo110217 22822473 PMC3399545

[B12] Chatzimanouil M. K. T. Wilkens L. Anders H.-J. (2018). Quantity and reporting quality of kidney research. *J. Am. Soc. Nephrol.* 30 13–22. 10.1681/ASN.2018050515 30545982 PMC6317598

[B13] Chau B. N. Xin C. Hartner J. Ren S. Castano A. P. Linn G. (2012). MicroRNA-21 promotes fibrosis of the kidney by silencing metabolic pathways. *Sci. Transl. Med.* 4:121ra18. 10.1126/scitranslmed.3003205 22344686 PMC3672221

[B14] Clements M. Gershenovich M. Chaber C. Campos-Rivera J. Du P. Zhang M. (2016). Differential Ly6C expression after renal ischemia-reperfusion identifies unique macrophage populations. *J. Am. Soc. Nephrol.* 27 159–170. 10.1681/ASN.2014111138 26015452 PMC4696575

[B15] Delella F. K. de Almeida F. L. A. Nunes H. C. Rinaldi J. C. Felisbino S. L. (2017). Fibrillar collagen genes are not coordinately upregulated with TGF β1 expression in finasteride-treated prostate. *Cell Biol. Int.* 41 1214–1222. 10.1002/cbin.10787 28493523

[B16] Denby L. Ramdas V. Lu R. Conway B. R. Grant J. S. Dickinson B. (2014). MicroRNA-214 antagonism protects against renal fibrosis. *J. Am. Soc. Nephrol.* 25 65–80. 10.1681/ASN.2013010072 24158985 PMC3871772

[B17] Denby L. Ramdas V. McBride M. W. Wang J. Robinson H. McClure J. (2011). miR-21 and miR-214 are consistently modulated during renal injury in rodent models. *Am. J. Pathol.* 179 661–672. 10.1016/j.ajpath.2011.04.021 21704009 PMC3157202

[B18] Di L. Andrew L. Antonio H. Santoboni A. Russo D. Ronco C. (2015). Chronic kidney disease and cardiovascular complications. *Hear. Fail Rev.* 20 259–272. 10.1007/s10741-014-9460-9 25344016

[B19] Dilauro M. Zimpelmann J. Robertson S. J. Genest D. Burns K. D. (2010). Effect of ACE2 and angiotensin-(1–7) in a mouse model of early chronic kidney disease. *Am J Physiol Ren. Physiol.* 298 F1523–F1532. 10.1152/ajprenal.00426.2009 20357030

[B20] Eardley K. S. Zehnder D. Quinkler M. Lepenies J. Bates R. L. Savage C. O. (2006). The relationship between albuminuria, MCP-1/CCL2, and interstitial macrophages in chronic kidney disease. *Kidney Int.* 69 1189–1197. 10.1038/sj.ki.5000212 16609683

[B21] Eckardt K. Coresh J. Devuyst O. Johnson R. J. Levey A. S. Levin A. (2013). Evolving importance of kidney disease: from subspecialty to global health burden. *Lancet* 382 158–169. 10.1016/S0140-6736(13)60439-0 23727165

[B22] Feldman H. I. Peralta C. A. Inker L. A. Lash J. P. Fox C. H. Kurella Tamura M. (2014). KDOQI US commentary on the 2012 KDIGO clinical practice guideline for the evaluation and management of CKD. *Am. J. Kidney Dis.* 63 713–735. 10.1053/j.ajkd.2014.01.416 24647050

[B23] Felsenfeld A. J. Levine B. S. Rodriguez M. (2015). Pathophysiology of calcium, phosphorus, and magnesium dysregulation in chronic kidney disease. *Semin. Dial.* 28 564–577. 10.1111/sdi.12411 26303319

[B24] Foltz C. J. Ullman-Culleré M. (1999). Guidelines for assessing the health and condition of mice. *Lab. Anim.* 28 28–32. 10403450

[B25] Fontes M. S. C. Papazova D. A. Van Koppen A. De Jong S. Korte S. M. Bongartz L. G. (2015). Arrhythmogenic remodeling in murine models of deoxycorticosterone acetate-salt-induced and 5/6-subtotal nephrectomy-salt-induced cardiorenal disease. *CardioRenal Med.* 5 208–218. 10.1159/000430475 26195973 PMC4478316

[B26] Gao S. Ho D. Vatner D. E. Vatner S. F. (2011). Murine echocardiography. *Curr. Protoc. Mouse Biol.* 1 71–83. 10.1002/9780470942390.mo100130 21743841 PMC3130310

[B27] Gava A. L. Freitas F. P. Balarini C. M. Vasquez E. C. Meyrelles S. S. (2012). Effects of 5/6 nephrectomy on renal function and blood pressure in mice. *Int. J. Physiol. Pathophysiol. Pharmacol.* 4 167–173. 23071874 PMC3466491

[B28] Gewin L. Zent R. Pozzi A. (2017). Progression of chronic kidney disease: too much cellular talk causes damage. *Kidney Int.* 91 552–560. 10.1016/j.kint.2016.08.025 27773427 PMC5313325

[B29] Gewin L. S. (2018). Renal fibrosis: primacy of the proximal tubule. *Matrix Biol.* 6 248–262. 10.1016/j.matbio.2018.02.006 29425694 PMC6015527

[B30] Gomez I. G. Mackenna D. A. Johnson B. G. Kaimal V. Roach A. M. Ren S. (2015). Anti–microRNA-21 oligonucleotides prevent Alport nephropathy progression by stimulating metabolic pathways. *J. Clin. Invest.* 125 141–156. 10.1172/jci75852 25415439 PMC4382246

[B31] Gosmanova E. O. Kovesdy C. P. (2016). Blood pressure targets in CKD: lessons learned from sprint and previous observational studies. *Curr. Cardiol. Rep.* 18:88. 10.1007/s11886-016-0769-y 27448402

[B32] Heerspink H. J. L. Gansevoort R. T. (2015). Albuminuria Is an appropriate therapeutic target in patients with CKD: the pro view. *Clin. J. Am. Soc. Nephrol.* 10 1079–1088. 10.2215/CJN.11511114 25887073 PMC4455219

[B33] Hennino M.-F. Buob D. Van der Hauwaert C. Gnemmi V. Jomaa Z. Pottier N. (2016). miR-21-5p renal expression is associated with fibrosis and renal survival in patients with IgA nephropathy. *Sci. Rep.* 6:27209. 10.1038/srep27209 27264483 PMC4893709

[B34] Hewitson T. D. (2009). Renal tubulointerstitial fibrosis: common but never simple. *Am. J. Physiol. Ren. Physiol.* 296 F1239–F1244. 10.1152/ajprenal.90521.2008 19144691

[B35] Hewitson T. D. Holt S. G. Smith E. R. (2015). Animal models to study links between cardiovascular disease and renal failure and their relevance to human pathology. *Front. Immunol.* 6:465. 10.3389/fimmu.2015.00465 26441970 PMC4585255

[B36] Hewitson T. D. Holt S. G. Smith E. R. (2017). Progression of tubulointerstitial fibrosis and the chronic kidney disease phenotype - role of risk factors and epigenetics. *Front. Pharmacol.* 8:520. 10.3389/fphar.2017.00520 28848437 PMC5550676

[B37] Hill N. R. Fatoba S. T. Oke J. L. Hirst J. A. O’Callaghan C. A. Lasserson D. S. (2016). Global prevalence of chronic kidney disease – a systematic review and meta-analysis. *PLoS One* 6:e0158765. 10.1371/journal.pone.0158765 27383068 PMC4934905

[B38] Horowitz B. Miskulin D. Zager P. (2015). Epidemiology of hypertension in CKD. *Adv. Chronic Kidney Dis.* 22 88–95. 10.1053/j.ackd.2014.09.004 25704344

[B39] Hyde G. D. Taylor R. F. Ashton N. Borland S. J. Wu H. S. G. Gilmore A. P. (2014). Axl tyrosine kinase protects against tubulo-interstitial apoptosis and progression of renal failure in a murine model of chronic kidney disease and hyperphosphataemia. *PLoS One* 9:e102096. 10.1371/journal.pone.0102096 25019319 PMC4096921

[B40] Ishola D. A. van der Giezen D. M. Hahnel B. Goldschmeding R. Kriz W. Koomans H. A. (2006). In mice, proteinuria and renal inflammatory responses to albumin overload are strain-dependent. *Nephrol. Dial. Transplant.* 21 591–597. 10.1093/ndt/gfi303 16326737

[B41] Ito K. Chen J. El Chaar M. Stern J. M. Seshan S. V. Khodadadian J. J. (2004). Renal damage progresses despite improvement of renal function after relief of unilateral ureteral obstruction in adult rats. *Am. J. Physiol. Ren. Physiol.* 287 F1283–F1293. 15328069 10.1152/ajprenal.00441.2003

[B42] Jha V. Garcia-Garcia G. Iseki K. Li Z. Naicker S. Plattner B. (2013). Chronic kidney disease: global dimension and perspectives. *Lancet* 382 260–272. 10.1016/S0140-6736(13)60687-X 23727169

[B43] Jones F. E. Bailey M. A. Murray L. S. Lu Y. Mcneilly S. Lennon R. (2016). ER stress and basement membrane defects combine to cause glomerular and tubular renal disease resulting from Col4a1 mutations in mice. *Dis. Model. Mech.* 9 165–176. 10.1242/dmm.021741 26839400 PMC4770143

[B44] Jörres A. Lemke H. D. Henle T. Descamps-Latscha B. De Smet R. Glorieux G. (2004). Review on uremic toxins: classification, concentration, and interindividual variability. *Kidney Int.* 63 1934–1943. 10.1046/j.1523-1755.2003.00924.x 12675874

[B45] Kennedy D. J. Elkareh J. Shidyak A. Shapiro A. P. Smaili S. Mutgi K. (2008). Partial nephrectomy as a model for uremic cardiomyopathy in the mouse. *Am. J. Physiol. Ren. Physiol.* 294 F450–F454. 18032546 10.1152/ajprenal.00472.2007 PMC2742580

[B46] Kerkelä R. Ulvila J. Magga J. (2015). Natriuretic peptides in the regulation of cardiovascular physiology and metabolic events. *J. Am. Heart Assoc.* 4 1–13. 10.1161/JAHA.115.002423 PMC4845118 26508744

[B47] Kerr M. Bray B. Medcalf J. O’Donoghue D. J. Matthews B. (2012). Estimating the financial cost of chronic kidney disease to the NHS in England. *Nephrol. Dial. Transplant.* 27(Suppl. 3):iii73–80. 10.1093/ndt/gfs269 22815543 PMC3484716

[B48] Kramann R. Schneider R. K. Dirocco D. P. Machado F. Fleig S. Bondzie P. A. (2015). Perivascular Gli1+ progenitors are key contributors to injury-induced organ fibrosis. *Cell Stem Cell* 16 51–66. 10.1016/j.stem.2014.11.004 25465115 PMC4289444

[B49] Kren S. Hostetter T. H. (1999). The course of the remnant kidney model in mice. *Kidney Int.* 56 333–337. 10.1046/j.1523-1755.1999.00527.x 10411710

[B50] Lacson E. Levin N. (2004). C-reactive protein and end-stage renal disease. *Semin. Dial.* 17 438–448. 15660574 10.1111/j.0894-0959.2004.17604.x

[B51] Lau W. L. Vaziri N. D. (2016). Urea, a true uremic toxin: the empire strikes back. *Clin. Sci.* 131 3–12. 10.1042/cs20160203 27872172

[B52] Leelahavanichkul A. Yan Q. Hu X. Eisner C. Huang Y. Chen R. (2010). Angiotensin II overcomes strain-dependent resistance of rapid CKD progression in a new remnant kidney mouse model. *Kidney Int.* 78 1136–1153. 10.1038/ki.2010.287 20736988 PMC3113489

[B53] Levey A. S. Inker L. A. Coresh J. (2014). GFR estimation: from physiology to public health. *Am. J. Kidney Dis.* 63 820–834. 10.1053/j.ajkd.2013.12.006 24485147 PMC4001724

[B54] Li K. L. He Y. N. Chen J. Zhan J. Li Z. H. Zhao L. (2012). P53 negatively regulates the osteogenic differentiation of vascular smooth muscle cells in mice with chronic kidney disease. *Cardiovasc. J. Afr.* 23 e1–e9. 10.5830/CVJA-2011-069 22143460 PMC3734878

[B55] Lin G. H. Y. Sedgmen B. J. Moraes T. J. Snell L. M. Topham D. J. Watts T. H. (2009). Endogenous 4-1BB ligand plays a critical role in protection from influenza-induced disease. *J. Immunol.* 182 934–947. 10.4049/jimmunol.182.2.934 19124736

[B56] Lindsey M. L. Kassiri Z. Virag J. A. I. de Castro Brás L. E. Scherrer-Crosbie M. (2018). Guidelines for measuring cardiac physiology in mice. *Am. J. Physiol. Circ. Physiol.* 314 H733–H752. 10.1152/ajpheart.00339.2017 29351456 PMC5966769

[B57] Liu S. (2019). Heart-kidney interactions: mechanistic insights from animal models. *Am. J. Physiol. Ren. Physiol.* 316 F974–F985. 10.1152/ajprenal.00624.2017 30838876

[B58] Locatelli F. Cannata-Andia J. B. Drueke T. B. Horl W. H. Fouque D. Heimburger O. (2002). Management of disturbances of calcium and phosphate metabolism in chronic renal insufficiency, with emphasis on the control of hyperphosphataemia. *Nephrol. Dial. Transplant.* 17 723–731. 10.1093/ndt/17.5.723 11981055

[B59] Lv W. Fan F. Wang Y. Gonzalez-Fernandez E. Wang C. Yang L. (2018). Therapeutic potential of microRNAs for the treatment of renal fibrosis and CKD. *Physiol. Genom.* 50 20–34. 10.1152/physiolgenomics.00039.2017 29127220 PMC5866411

[B60] Ma L.-J. Fogo A. B. (2003). Model of robust induction of glomerulosclerosis in mice: importance of genetic background. *Kidney Int.* 64 350–355. 10.1046/j.1523-1755.2003.00058.x 12787428

[B61] Mack M. Yanagita M. (2015). Origin of myofibroblasts and cellular events triggering fibrosis. *Kidney Int.* 87 297–307. 10.1038/ki.2014.287 25162398

[B62] Martin K. J. González E. A. (2011). Prevention and control of phosphate retention/hyperphosphatemia in CKD-MBD: what is normal, when to start, and how to treat? *Clin. J. Am. Soc. Nephrol.* 6 440–446. 10.2215/CJN.05130610 21292848

[B63] Meyer T. W. Anderson S. Rennke H. G. Brenner B. M. (1985). Converting enzyme inhibitor therapy limits progressive glomerular injury in rats with renal insufficiency. *Am. J. Med.* 79 31–36. 10.1016/0002-9343(85)90077-4 2996344

[B64] Murray P. J. Allen J. E. Biswas S. K. Fisher E. A. Gilroy D. W. Goerdt S. (2014). Macrophage activation and polarization: nomenclature and experimental guidelines. *Immunity* 41 14–20. 10.1016/j.immuni.2014.06.008 25035950 PMC4123412

[B65] Nakagawa N. Duffield J. S. (2013). Myofibroblasts in fibrotic kidneys. *Curr. Pathobiol. Rep.* 1 189–198. 10.1007/s40139-013-0025-8 24187654 PMC3810972

[B66] Nakagawa S. Nishihara K. Miyata H. Shinke H. Tomita E. Kajiwara M. (2015). Molecular markers of tubulointerstitial fibrosis and tubular cell damage in patients with chronic kidney disease. *PLoS One* 10:e0136994. 10.1371/journal.pone.0136994 26317775 PMC4552842

[B67] Nath K. A. (1992). Tubulointerstitial changes as a major determinant in the progression of renal damage. *Am. J. Kidney Dis.* 20 1–17. 10.1016/s0272-6386(12)80312-x 1621674

[B68] Obrador G. T. Levin A. (2019). CKD hotspots: challenges and areas of opportunity. *Semin. Nephrol.* 39 308–314. 10.1016/j.semnephrol.2019.02.009 31054631

[B69] Oosterhuis N. R. Papazova D. A. Gremmels H. Joles J. A. Verhaar M. C. (2017). T-cells contribute to hypertension but not to renal injury in mice with subtotal nephrectomy. *BMC Nephrol.* 18:153. 10.1186/s12882-017-0555-0 28482823 PMC5422945

[B70] Panichi V. Migliori M. De Pietro S. Taccola D. Bianchi A. M. Norpoth M. (2001). C reactive protein in patients with chronic renal diseases. *Ren. Fail.* 23 551–562. 10.1081/jdi-100104737 11499569

[B71] Perkins B. A. Ficociello L. H. Ostrander B. E. Silva K. H. Weinberg J. Warram J. H. (2007). Microalbuminuria and the risk for early progressive renal function decline in type 1 diabetes. *J. Am. Soc. Nephrol.* 18 1353–1361. 10.1681/asn.2006080872 17329575

[B72] Peters R. Staessen J. A. Cheung A. K. Beckett N. Nadkarni G. N. Mant J. (2017). Association between more intensive vs less intensive blood pressure lowering and risk of mortality in chronic kidney disease stages 3 to 5. *JAMA Intern. Med.* 177:1498. 10.1001/jamainternmed.2017.4377 28873137 PMC5704908

[B73] Purnomo E. Emoto N. Nugrahaningsih D. A. A. Nakayama K. Yagi K. Heiden S. (2013). Glycosaminoglycan overproduction in the aorta increases aortic calcification in murine chronic kidney disease. *J. Am. Heart Assoc.* 2 1–18. 10.1161/JAHA.113.000405 23985378 PMC3835254

[B74] Ramachandran P. Pellicoro A. Vernon M. A. Boulter L. Aucott R. L. Ali A. (2012). Differential Ly-6C expression identifies the recruited macrophage phenotype, which orchestrates the regression of murine liver fibrosis. *Proc. Natl. Acad. Sci. U.S.A.* 109 E3186–E3195. 10.1073/pnas.1119964109 23100531 PMC3503234

[B75] Rambausek M. Ritz E. Mall G. Mehls O. Katus H. (1985). Myocardial hypertrophy in rats with renal insufficiency. *Kidney Int.* 28 775–782. 10.1038/ki.1985.197 2935673

[B76] Respress J. L. Wehrens X. H. T. (2010). Transthoracic echocardiography in mice. *J. Vis. Exp.* 39:1738. 10.3791/1738 20517201 PMC3144600

[B77] Riaz N. Wolden S. L. Gelblum D. Y. Eric J. (2015). Atrial natriuretic peptide in cardiovascular biology and disease (NPPA). *Gene* 569 1–6. 10.1016/j.gene.2015.06.029 26074089 PMC4496260

[B78] Ritter C. S. Slatopolsky E. (2016). Phosphate toxicity in CKD: the killer among us. *Clin. J. Am. Soc. Nephrol.* 11 1088–1100. 10.2215/CJN.11901115 26912542 PMC4891758

[B79] Rosendahl A. Kabiri R. Bode M. Cai A. Klinge S. Ehmke H. (2018). Adaptive immunity and IL-17A are not involved in the progression of chronic kidney disease after 5/6 nephrectomy in mice. *Br. J. Pharmacol.* 176 2002–2014. 10.1111/bph.14509 30270435 PMC6534810

[B80] Schelling J. R. (2016). Tubular atrophy in the pathogenesis of chronic kidney disease progression. *Pediatr. Nephrol.* 31 693–706. 10.1007/s00467-015-3169-4 26208584 PMC4726480

[B81] Schnelle M. Catibog N. Zhang M. Nabeebaccus A. A. Anderson G. Richards D. A. (2018). Echocardiographic evaluation of diastolic function in mouse models of heart disease. *J. Mol. Cell. Cardiol.* 114 20–28. 10.1016/j.yjmcc.2017.10.006 29055654 PMC5807035

[B82] Siedlecki A. M. Jin X. Muslin A. J. (2009). Uremic cardiac hypertrophy is reversed by rapamycin but not by lowering of blood pressure. *Kidney Int.* 75 800–808. 10.1038/ki.2008.690 19165175 PMC2764402

[B83] Soler M. J. Wysocki J. Batlle D. (2008). Angiotensin-converting enzyme 2 and the kidney. *Exp. Physiol.* 93 549–556. 18223023 10.1113/expphysiol.2007.041350

[B84] Song S. Meyer M. Türk T. R. Wilde B. Feldkamp T. Assert R. (2009). Serum cystatin C in mouse models: a reliable and precise marker for renal function and superior to serum creatinine. *Nephrol. Dial. Transplant.* 24 1157–1161. 10.1093/ndt/gfn626 19004848

[B85] Stevens P. E. Levin A. (2013). Evaluation and management of chronic kidney disease: synopsis of the kidney disease: improving global outcomes 2012 clinical practice guideline. *Ann. Intern. Med.* 158 825–830. 10.7326/0003-4819-158-11-201306040-00007 23732715

[B86] Tan R. Z. Zhong X. Li J. C. Zhang Y. W. Yan Y. Liao Y. (2019). An optimized 5/6 nephrectomy mouse model based on unilateral kidney ligation and its application in renal fibrosis research. *Ren. Fail.* 41 555–566. 10.1080/0886022X.2019.1627220 31234688 PMC6598497

[B87] Taylor P. R. Martinez-Pomares L. Stacey M. Lin H.-H. Brown G. D. Gordon S. (2005). Macrophage receptors and immune recognition. *Annu. Rev. Immunol.* 23 901–944. 10.1146/annurev.immunol.23.021704.115816 15771589

[B88] Ucero A. C. Benito-Martin A. Izquierdo M. C. Sanchez-Niño M. D. Sanz A. B. Ramos A. M. (2014). Unilateral ureteral obstruction: beyond obstruction. *Int. Urol. Nephrol.* 46 765–776. 10.1007/s11255-013-0520-1 24072452

[B89] Ullman-Culleré M. H. Foltz C. J. (1999). Body condition scoring: a rapid and accurate method for assessing health status in mice. *Lab. Anim. Sci.* 49 319–323. 10403450

[B90] Vanholder R. Gryp T. Glorieux G. (2018). Urea and chronic kidney disease: the comeback of the century? (in uraemia research). *Nephrol. Dial. Transplant.* 33 4–12. 10.1093/ndt/gfx039 28407121

[B91] Vassalotti J. A. Centor R. Turner B. J. Greer R. C. Choi M. Sequist T. D. (2016). Practical approach to detection and management of chronic kidney disease for the primary care clinician. *Am. J. Med.* 129:153-162.e7. 10.1016/j.amjmed.2015.08.025 26391748

[B92] Vavrinec P. Buikema H. Vettoretti S. Deelman L. E. De Zeeuw D. Henning R. H. (2016). Renal endothelial function is associated with the anti-proteinuric effect of ACE inhibition in 5/6 nephrectomized rats. *Am. J. Physiol. Physiol.* 310 F1047–F1053. 10.1152/ajprenal.00325.2015 26911850

[B93] Venkatachalam M. A. Weinberg J. M. Kriz W. Bidani A. K. (2015). Failed tubule recovery, AKI-CKD transition, and kidney disease progression. *J. Am. Soc. Nephrol.* 26 1765–1776. 10.1681/ASN.2015010006 25810494 PMC4520181

[B94] Vervloet M. G. Sezer S. Massy Z. A. Johansson L. Cozzolino M. Fouque D. (2017). The role of phosphate in kidney disease. *Nat. Rev. Nephrol.* 13 27–38. 10.1038/nrneph.2016.164 27867189

[B95] Waheed S. Matsushita K. Sang Y. Hoogeveen R. Ballantyne C. Coresh J. (2012). Combined association of albuminuria and cystatin C-based estimated GFR with mortality, coronary heart disease, and heart failure outcomes: the atherosclerosis risk in communities (ARIC) study. *Am. J. Kidney Dis.* 60 207–216. 10.1053/j.ajkd.2012.03.011 22537422 PMC3582350

[B96] Wang Y. Thatcher S. E. Cassis L. (2017). Measuring blood pressure using a noninvasive tail cuff method in mice. *Methods Mol. Biol.* 1614 69–73. 10.1007/978-1-4939-7030-8_6 28500596

[B97] Webster A. C. Nagler E. V. Morton R. L. Masson P. (2017). Chronic kidney disease. *Lancet* 389 1238–1252. 10.1016/S0140-6736(16)32064-5 27887750

[B98] Windt W. A. K. M. van Dokkum R. P. E. Kluppel C. A. Jeronimus-Stratingh C. M. Hut F. de Zeeuw D. (2008). Therapeutic resistance to angiotensin converting enzyme (ACE) inhibition is related to pharmacodynamic and -kinetic factors in 5/6 nephrectomized rats. *Eur. J. Pharmacol.* 580 231–240. 10.1016/j.ejphar.2007.10.060 18036585

[B99] Yang H.-C. Zuo Y. Fogo A. B. (2010). Models of chronic kidney disease. *Drug Discov. Today Dis. Model.* 7 13–19. 10.1016/j.ddmod.2010.08.002 PMC3030258 21286234

[B100] Zeng L. Mathew A. V. Byun J. Atkins K. B. Brosius F. C. Pennathur S. (2018). Myeloperoxidase-derived oxidants damage artery wall proteins in an animal model of chronic kidney disease–accelerated atherosclerosis. *J. Biol. Chem.* 293 7238–7249. 10.1074/jbc.ra117.000559 29581235 PMC5949994

