# Supplementary material for: Refining the Mouse Subtotal Nephrectomy in Male 129S2/SV Mice for Consistent Modeling of Progressive Kidney Disease With Renal Inflammation and Cardiac Dysfunction
Source: Front Physiol. 2019 Nov 15;10:1365. doi: 10.3389/fphys.2019.01365 (PMC6872545; doi:10.3389/fphys.2019.01365)
Supplement: Supplementary file 1 [file Presentation_1.pptx]

## Slide 1
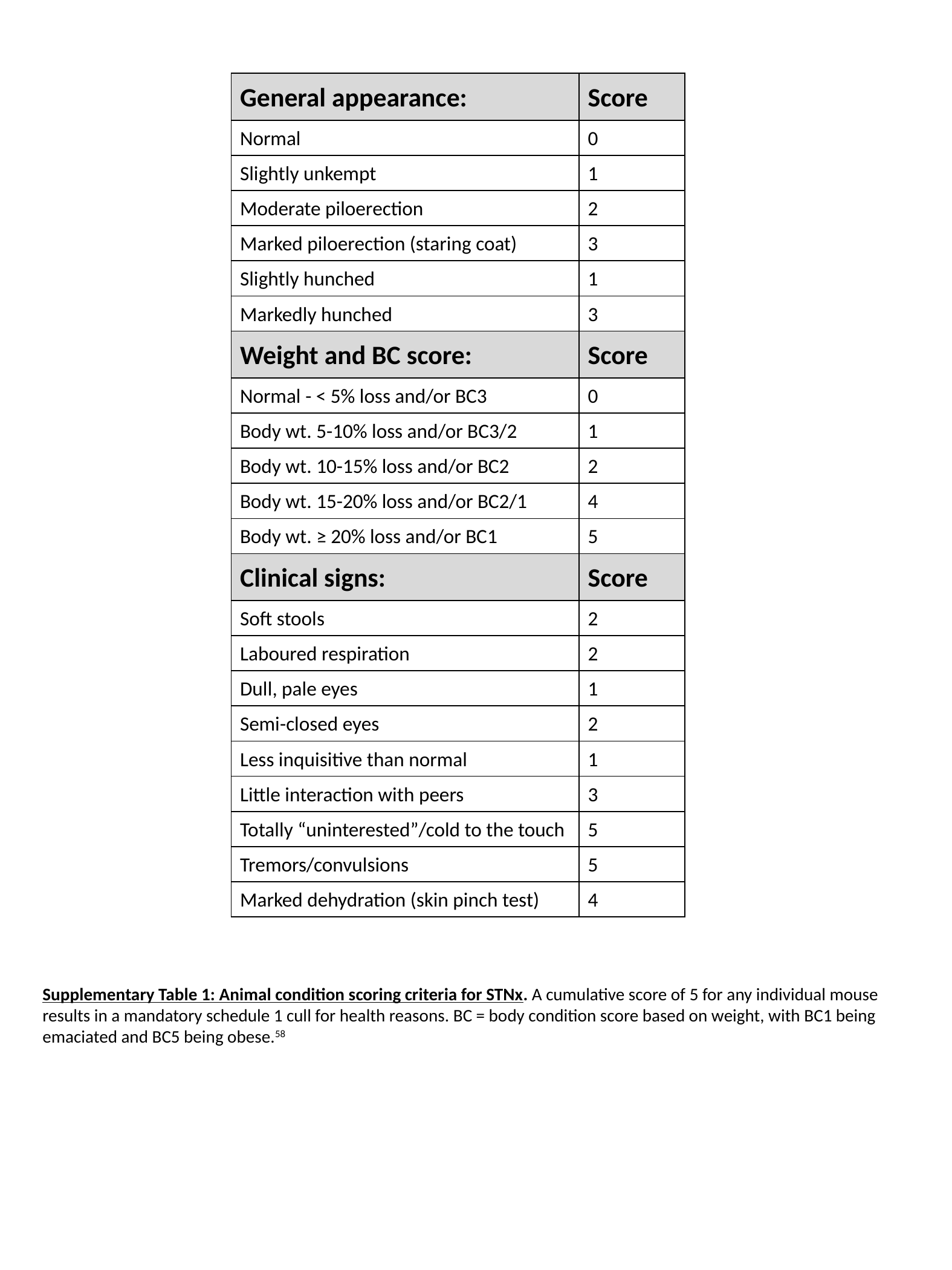

| General appearance: | Score |
| --- | --- |
| Normal | 0 |
| Slightly unkempt | 1 |
| Moderate piloerection | 2 |
| Marked piloerection (staring coat) | 3 |
| Slightly hunched | 1 |
| Markedly hunched | 3 |
| Weight and BC score: | Score |
| Normal - < 5% loss and/or BC3 | 0 |
| Body wt. 5-10% loss and/or BC3/2 | 1 |
| Body wt. 10-15% loss and/or BC2 | 2 |
| Body wt. 15-20% loss and/or BC2/1 | 4 |
| Body wt. ≥ 20% loss and/or BC1 | 5 |
| Clinical signs: | Score |
| Soft stools | 2 |
| Laboured respiration | 2 |
| Dull, pale eyes | 1 |
| Semi-closed eyes | 2 |
| Less inquisitive than normal | 1 |
| Little interaction with peers | 3 |
| Totally “uninterested”/cold to the touch | 5 |
| Tremors/convulsions | 5 |
| Marked dehydration (skin pinch test) | 4 |
Supplementary Table 1: Animal condition scoring criteria for STNx. A cumulative score of 5 for any individual mouse results in a mandatory schedule 1 cull for health reasons. BC = body condition score based on weight, with BC1 being emaciated and BC5 being obese.58

## Slide 2
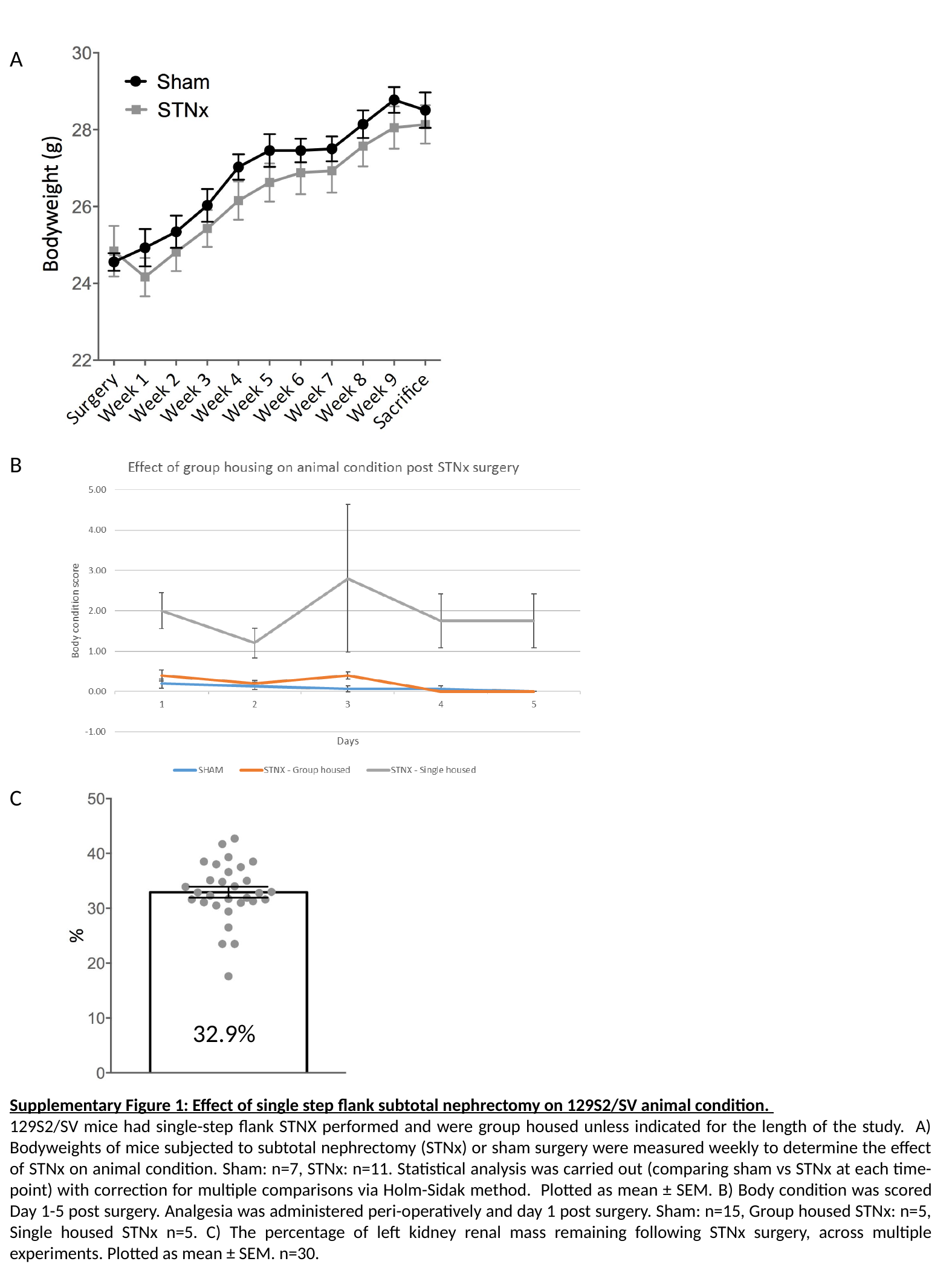

A
B
C
32.9%
Supplementary Figure 1: Effect of single step flank subtotal nephrectomy on 129S2/SV animal condition.
129S2/SV mice had single-step flank STNX performed and were group housed unless indicated for the length of the study. A) Bodyweights of mice subjected to subtotal nephrectomy (STNx) or sham surgery were measured weekly to determine the effect of STNx on animal condition. Sham: n=7, STNx: n=11. Statistical analysis was carried out (comparing sham vs STNx at each time-point) with correction for multiple comparisons via Holm-Sidak method. Plotted as mean ± SEM. B) Body condition was scored Day 1-5 post surgery. Analgesia was administered peri-operatively and day 1 post surgery. Sham: n=15, Group housed STNx: n=5, Single housed STNx n=5. C) The percentage of left kidney renal mass remaining following STNx surgery, across multiple experiments. Plotted as mean ± SEM. n=30.

## Slide 3
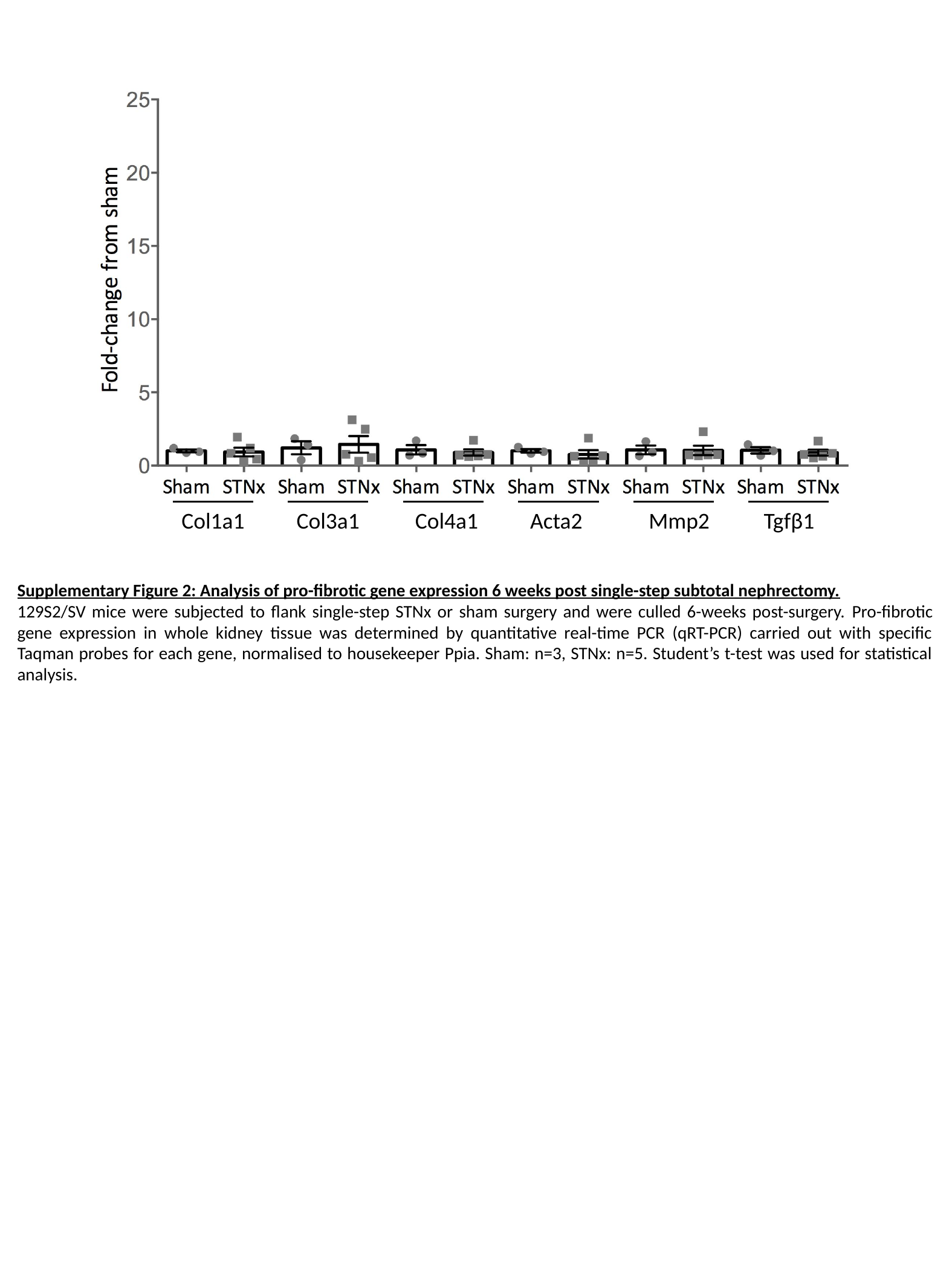

Col1a1
Col4a1
Mmp2
Col3a1
Acta2
Tgfβ1
Supplementary Figure 2: Analysis of pro-fibrotic gene expression 6 weeks post single-step subtotal nephrectomy.
129S2/SV mice were subjected to flank single-step STNx or sham surgery and were culled 6-weeks post-surgery. Pro-fibrotic gene expression in whole kidney tissue was determined by quantitative real-time PCR (qRT-PCR) carried out with specific Taqman probes for each gene, normalised to housekeeper Ppia. Sham: n=3, STNx: n=5. Student’s t-test was used for statistical analysis.

## Slide 4
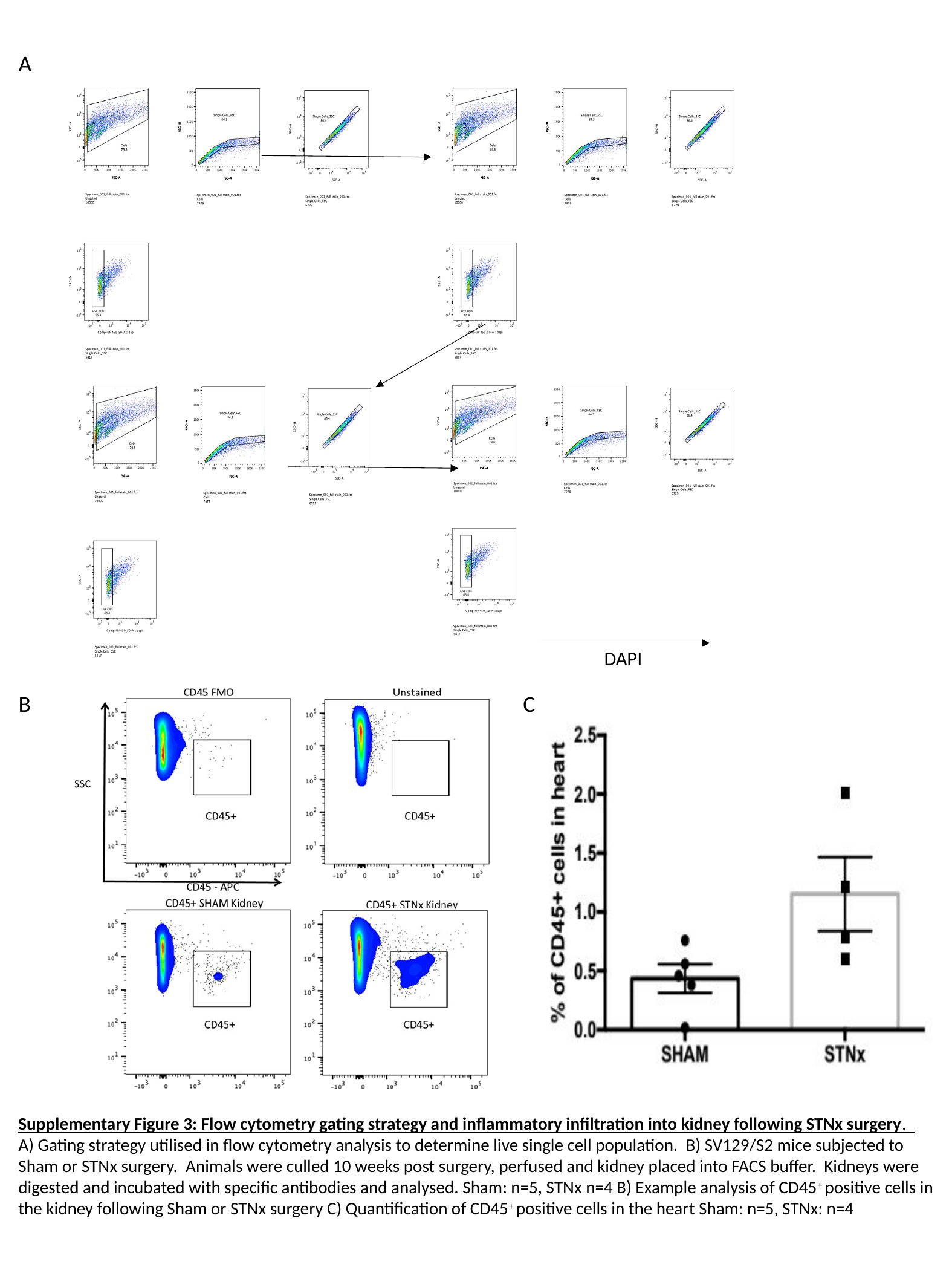

A
DAPI
B
C
Supplementary Figure 3: Flow cytometry gating strategy and inflammatory infiltration into kidney following STNx surgery.
A) Gating strategy utilised in flow cytometry analysis to determine live single cell population. B) SV129/S2 mice subjected to Sham or STNx surgery. Animals were culled 10 weeks post surgery, perfused and kidney placed into FACS buffer. Kidneys were digested and incubated with specific antibodies and analysed. Sham: n=5, STNx n=4 B) Example analysis of CD45+ positive cells in the kidney following Sham or STNx surgery C) Quantification of CD45+ positive cells in the heart Sham: n=5, STNx: n=4

## Slide 5
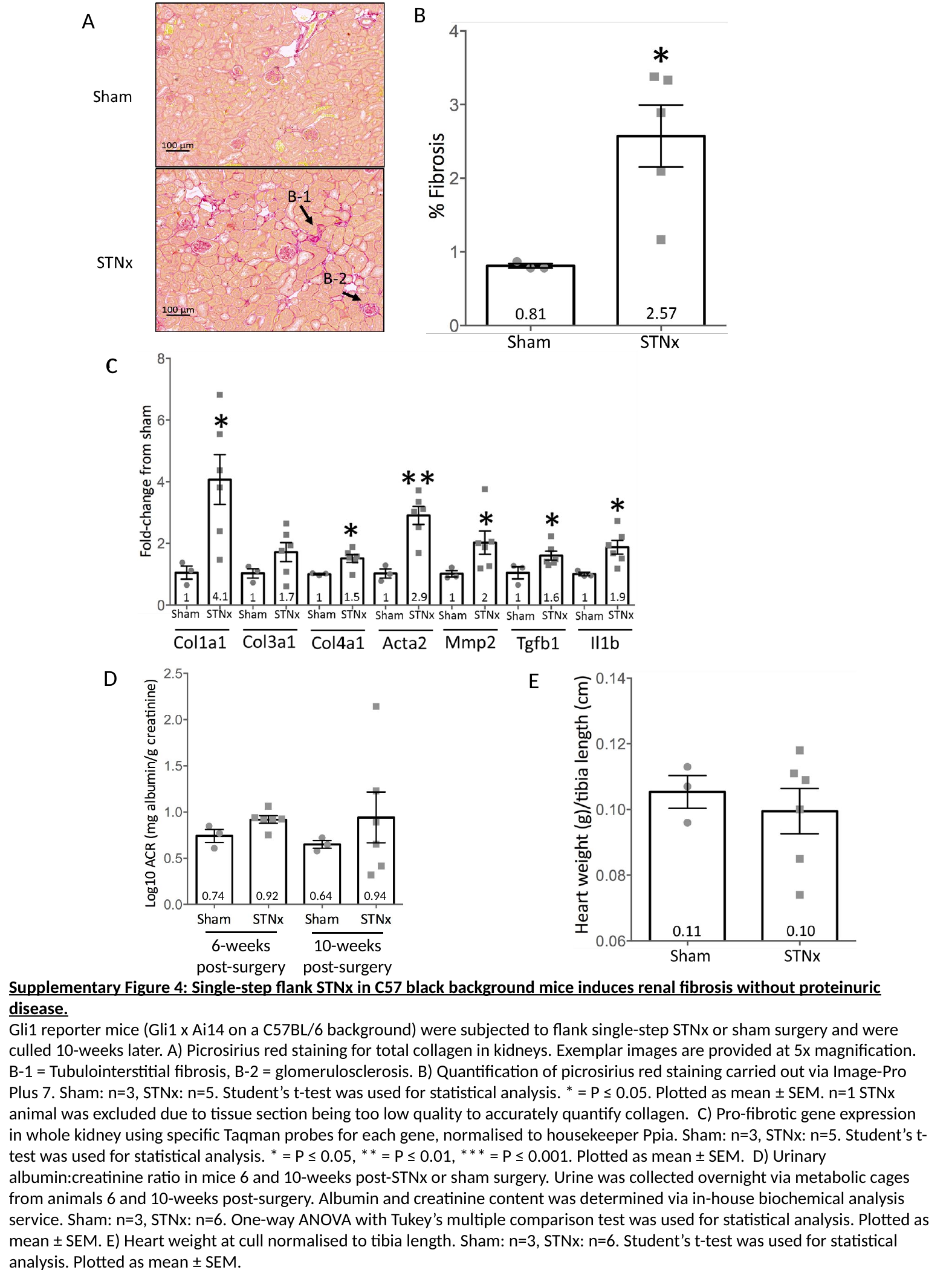

B
A
C
D
E
0.74
0.92
0.64
0.94
6-weeks
post-surgery
10-weeks
post-surgery
Supplementary Figure 4: Single-step flank STNx in C57 black background mice induces renal fibrosis without proteinuric disease.
Gli1 reporter mice (Gli1 x Ai14 on a C57BL/6 background) were subjected to flank single-step STNx or sham surgery and were culled 10-weeks later. A) Picrosirius red staining for total collagen in kidneys. Exemplar images are provided at 5x magnification. B-1 = Tubulointerstitial fibrosis, B-2 = glomerulosclerosis. B) Quantification of picrosirius red staining carried out via Image-Pro Plus 7. Sham: n=3, STNx: n=5. Student’s t-test was used for statistical analysis. * = P ≤ 0.05. Plotted as mean ± SEM. n=1 STNx animal was excluded due to tissue section being too low quality to accurately quantify collagen. C) Pro-fibrotic gene expression in whole kidney using specific Taqman probes for each gene, normalised to housekeeper Ppia. Sham: n=3, STNx: n=5. Student’s t-test was used for statistical analysis. * = P ≤ 0.05, ** = P ≤ 0.01, *** = P ≤ 0.001. Plotted as mean ± SEM. D) Urinary albumin:creatinine ratio in mice 6 and 10-weeks post-STNx or sham surgery. Urine was collected overnight via metabolic cages from animals 6 and 10-weeks post-surgery. Albumin and creatinine content was determined via in-house biochemical analysis service. Sham: n=3, STNx: n=6. One-way ANOVA with Tukey’s multiple comparison test was used for statistical analysis. Plotted as mean ± SEM. E) Heart weight at cull normalised to tibia length. Sham: n=3, STNx: n=6. Student’s t-test was used for statistical analysis. Plotted as mean ± SEM.
